# Transport of β-amyloid from brain to eye causes retinal degeneration in Alzheimer’s disease

**DOI:** 10.1084/jem.20240386

**Published:** 2024-09-24

**Authors:** Qiuchen Cao, Shige Yang, Xiaowei Wang, Huaiqing Sun, Weijie Chen, Yuliang Wang, Junying Gao, Yanchi Wu, Qiuhua Yang, Xue Chen, Songtao Yuan, Ming Xiao, Maiken Nedergaard, Yuqing Huo, Qinghuai Liu

**Affiliations:** 1 Department of Ophthalmology, The First Affiliated Hospital of Nanjing Medical University, Nanjing, China; 2 Jiangsu Province Key Laboratory of Neurodegeneration, Nanjing Medical University, Nanjing, China; 3 Department of Ophthalmology, Baylor College of Medicine, Houston, TX, USA; 4 Department of Cellular Biology and Anatomy, Vascular Biology Center, Medical College of Georgia, Augusta University, Augusta, GA, USA; 5 Faculty of Medical and Health Sciences, Center for Translational Neuromedicine, University of Copenhagen, Copenhagen, Denmark; 6 Department of Neurosurgery, Center for Translational Neuromedicine, University of Rochester Medical Center, Rochester, NY, USA; 7 Department of Neurology, The First Affiliated Hospital of Nanjing Medical University, Nanjing, China; 8 Department of Immunology, Key Laboratory of Immune Microenvironment and Diseases, Nanjing Medical University, Nanjing, China; 9 Nanjing Brain Hospital, Brain Institute, Nanjing Medical University, Nanjing, China

## Abstract

The eye is closely connected to the brain, providing a unique window to detect pathological changes in the brain. In this study, we discovered β-amyloid (Aβ) deposits along the ocular glymphatic system in patients with Alzheimer’s disease (AD) and 5×FAD transgenic mouse model. Interestingly, Aβ from the brain can flow into the eyes along the optic nerve through cerebrospinal fluid (CSF), causing retinal degeneration. Aβ is mainly observed in the optic nerve sheath, the neural axon, and the perivascular space, which might represent the critical steps of the Aβ transportation from the brain to the eyes. Aquaporin-4 facilitates the influx of Aβ in brain–eye transport and out-excretion of the retina, and its absence or loss of polarity exacerbates brain-derived Aβ induced damage and visual impairment. These results revealed brain-to-eye Aβ transport as a major contributor to AD retinopathy, highlighting a new therapeutic avenue in ocular and neurodegenerative disease.

## Teaser

The article identifies a direct connection between the brain and the eyes where Aβ from the brain can be transported to the eyes via the brain–eye pathway, leading to Alzheimer’s disease retinopathy.

## Introduction

Increasing evidence indicates that the pathological changes associated with Alzheimers disease (AD) are not limited to the brain but also extend to other organs such as the liver, kidneys, and intestines (Arnold et al., 2010; Huang et al., 2022; Wang et al., 2017). Individuals with AD frequently experience visual impairments, such as atypical pupillary responses, decreased contrast sensitivity, and visual field defects (Berisha et al., 2007). These visual abnormalities in AD patients are accompanied by various retinal pathological damages, including the degeneration of retinal ganglion cells, activation of glial cells, and narrowing of retinal blood vessels (Doustar et al., 2017; Frost et al., 2013; Hart et al., 2016; Koronyo et al., 2017). Furthermore, studies have documented the presence of β-amyloid (Aβ) plaques in the postmortem retina of AD patients and AD transgenic mouse models (Koronyo-Hamaoui et al., 2011; Koronyo et al., 2017; Shi et al., 2020).

The eye is part of the central nervous system (CNS) and shares the developmental origin with the brain. The optic nerve is surrounded by three layers of meninges that are continuous from those of the brain. The brain utilizes a unique perivascular network, the glymphatic system, to remove neurotoxic molecules, including Aβ (Iliff et al., 2012). Using an intravitreal infusion of multiple tracers, including human Aβ (hAβ), our recent studies identified an ocular glymphatic clearance system for the retinal waste product to exit via the proximal optic nerve (Wang et al., 2020). Interestingly, both the brain and the ocular glymphatic systems are dependent on glial water channel aquaporin-4 (AQP4), which mediates rapid fluid transmembrane transport and water homeostasis in the CNS. Despite the discovery of the ocular glymphatic system, little is known about its role in AD-related retinal degeneration. In this study, we investigated the retinopathy caused by Aβ in AD patients or 5×FAD mice, elucidating the role of the AQP4-mediated ocular glymphatic system in the retinal degeneration induced by brain-derived Aβ.

## Results

### Aβ deposition in the ocular blood vessels and periorbital lymphatic vessels of AD patients and mouse models

**Figure 1.**
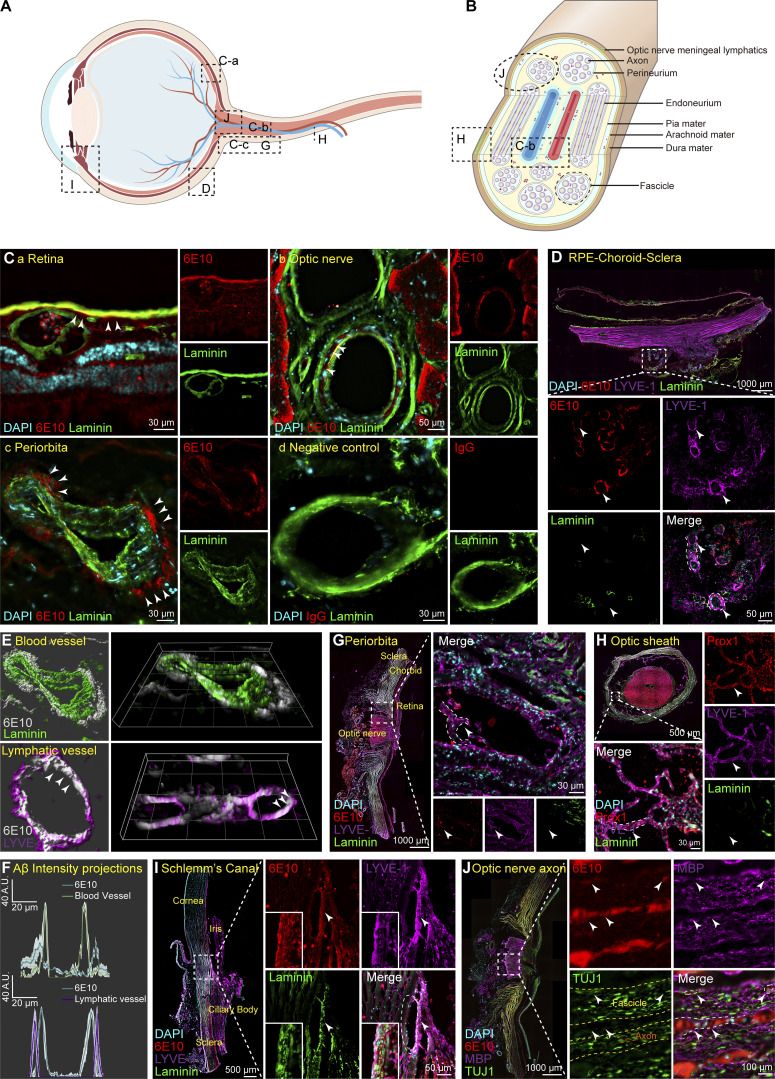
**Aβ deposits in the retina, optic nerve vasculature, and periorbital lymphatics of AD patients. (A and B)** The schematic diagram of the retina and optic nerve showing the distribution of blood vessels, lymphatics, and myelinated axons. **(C)** The retina and optic nerve sections were stained with 6E10 (red) for Aβ, and with Laminin (green) for retinal vessels (a), the central retinal vessel of the optic nerve (b), and periorbital blood vessels (c), with IgG used as a negative control (d). White arrowheads indicated the deposition of Aβ in the microvasculature of the retina, the intermediate layer of the vascular wall, and outside the vascular wall. **(D)** Representative immunofluorescence images of AD patient eye tissue sections stained with 6E10 (red), LYVE1 (purple), and Laminin (green) showing Aβ deposition in the posterior eye RPE-choroid-sclera complex. **(E and F)** The 3D reconstructed images and fluorescence intensity line graphs demonstrate the relationship between the deposition location of Aβ and the positions of blood vessels and lymphatic vessels. **(G–I)** Representative immunofluorescence images of AD patient eye tissue sections stained with 6E10 (red), LYVE1 (purple), and Laminin (green) showing Aβ deposition in the (G) periorbital lymphatics, and (H) optic nerve meningeal lymphatics, as well as (I) Schlemm’s canal. Prox1 (red) and Lyve1 (purple) were used to label lymphatic vessels. White arrowheads indicated Aβ deposition within the lumen of the lymphatic vessels. **(J)** Representative immunofluorescence images of AD patient eye tissue sections stained with 6E10 (red), MBP (purple), and TUJ1 (green) demonstrating Aβ deposition in the spaces between myelinated axons and fascicles.

AD patients exhibit visual defects, Aβ deposits, and vascular-related pathological changes in the retina (Koronyo et al., 2023; Shi et al., 2023). Recent reports indicate that Aβ primarily accumulates in the innermost layers of the retina, including the ganglion cell layer, inner plexiform layer, and inner nuclear layer, and the distribution of Aβ in the retina often correlates with blood vessels (Koronyo et al., 2017; Shi et al., 2020). However, the results regarding the location of Aβ deposition vary among different studies (Table S1). Therefore, using immunostaining with Aβ antibody 6E10, we first examined the deposition and distribution of Aβ in ocular and periocular areas of AD post-mortem eye tissues, including the retina, optic nerve, retinal pigment epithelium (RPE)-choroid-sclera complex, periocular tissues, and the aqueous outflow pathway (Fig. 1, A and B). Confocal imaging revealed that Aβ deposits were predominantly localized in the perivascular spaces (PVS) along the ophthalmic vasculatures, including retinal vessels, periorbital vessels, and central veins of the optic nerve (Fig. 1 C). In addition, the presence of ocular lymphatic vessels has been reported in recent years (Wang et al., 2020; Yin et al., 2024), although it remains a topic of debate (Uddin and Rutar, 2022). We found lymphatic-like vessels expressing prospero homeobox protein 1 (PROX1) and lymphatic vascular endothelial hyaluronan receptor 1 (LYVE1) but not Laminin in periocular tissues and the optic nerve sheath of AD patients. Notably, Aβ deposition was detected within these lymphatic vessels (Fig. 1, D–H), and 3D reconstructed images and fluorescence intensity line graphs showed that the Aβ deposits were located outside the vessel walls and inside the lymphatic vessels (Fig. 1, E and F). Aβ accumulation was also observed in the aqueous outflow pathway, mainly in the trabecular meshwork and Schlemm’s canal (Fig. 1 I), and in the axonal compartment of the optic nerve (Fig. 1 J).

**Figure 2.**
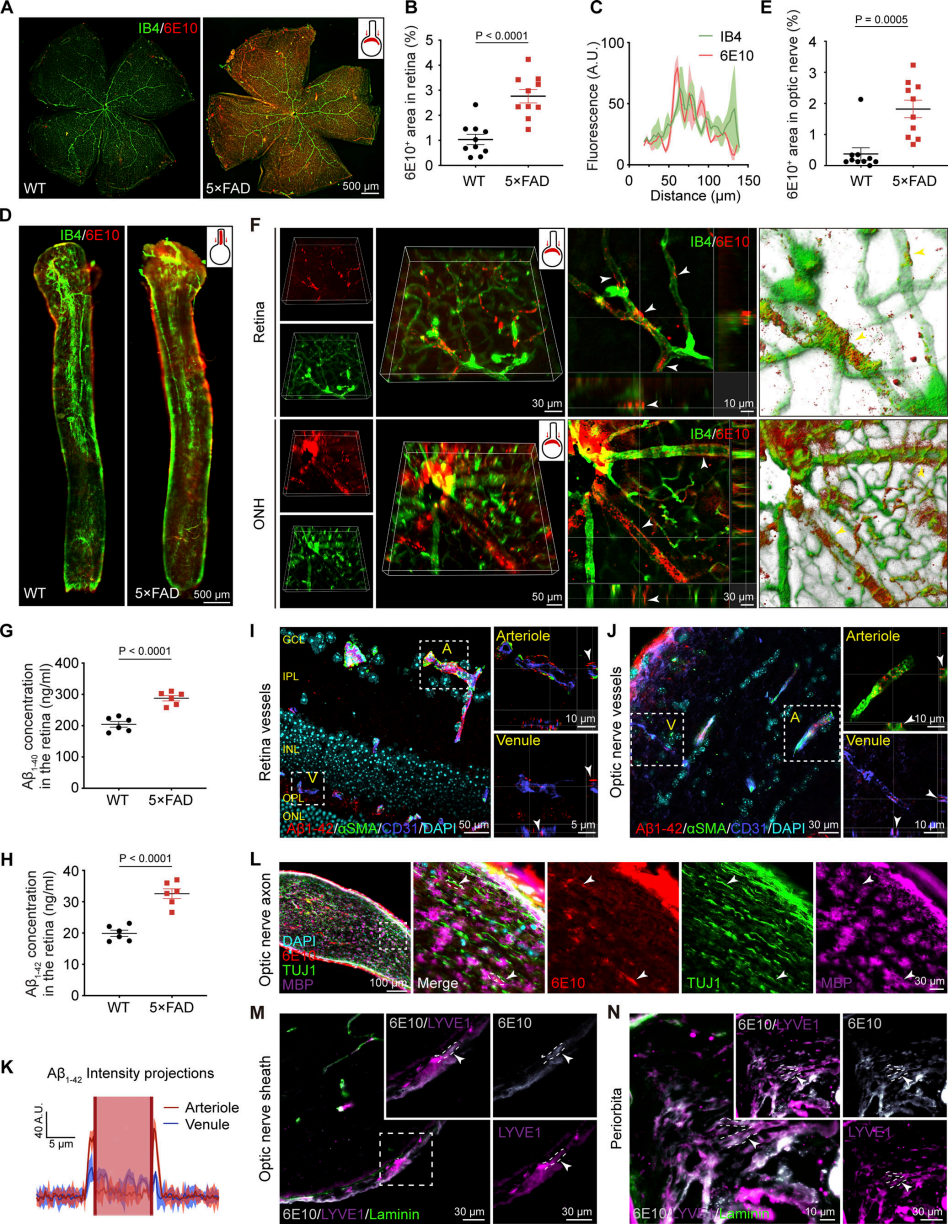
**Aβ deposition in ocular blood vessels and optic nerve meningeal lymphatic vessels of 5×FAD mice. (A and D)** Representative confocal images of retinal and optic nerve flatmounts from 10-mo-old WT and 5×FAD mice stained with 6E10 (red) for Aβ and IB4 (green) for blood vessels. **(B and E)** Statistical graphs showing the percentage of 6E10^+^ area in the retina and optic nerve (*n* = 10). **(C)** Fluorescence intensity profile through a line scan across retinal blood vessels, demonstrating co-localization of 6E10 and IB4. **(F)** 3D reconstruction of retina and optic nerve head (ONH) immunofluorescence pictures of 5×FAD mice labeled with 6E10 and IB4. White arrowheads indicate the majority of Aβ in the PVS. **(G and H)** ELISA of Aβ_1–40_ and Aβ_1–42_ in retinal samples from 10-mo-old WT mice and 5×FAD mice (*n* = 6). **(I and J)** Immunofluorescent staining of retina and optic nerve frozen sections from 5×FAD mice reveals Aβ_1–42_ (red) deposition at blood vessels, with αSMA (green)-positive and CD31 (blue)-positive labeling indicating arteries (A), and CD31 (blue) -positive and αSMA (green)-negative labeling indicating veins (V). **(K)** Fluorescence intensity line graph indicated that Aβ deposition in the PVS adjacent to arterioles was greater than that near venules, while deposition within the walls of venules was greater than inside the arterioles (*n* = 6–7). **(L)** Representative immunofluorescence images of 5×FAD mouse optic nerve tissue sections stained with 6E10 (red), MBP (purple), and TUJ1 (green). Aβ deposits in the spaces between myelinated axons were indicated by the arrowheads. **(M and N)** Representative immunofluorescence images showing Aβ labeled deposition with 6E10 (gray) in the optic nerve meningeal lymphatics and periorbital lymphatics in 5×FAD mice. The arrowheads indicated Aβ (gray) deposits colocalized with LYVE1^+^ (purple) and Laminin^−^ (green) labeled lymphatic vessels. Data are representative of two independent experiments. Data are presented as mean ± SEM. Statistical analysis was performed using two-tailed unpaired *t* tests (B, E, G, and H). GCL, ganglion cell layer; IPL, inner plexiform layer; INL, inner nuclear layer; OPL, outer plexiform layer; ONL, outer nuclear layer.

**Figure 3.**
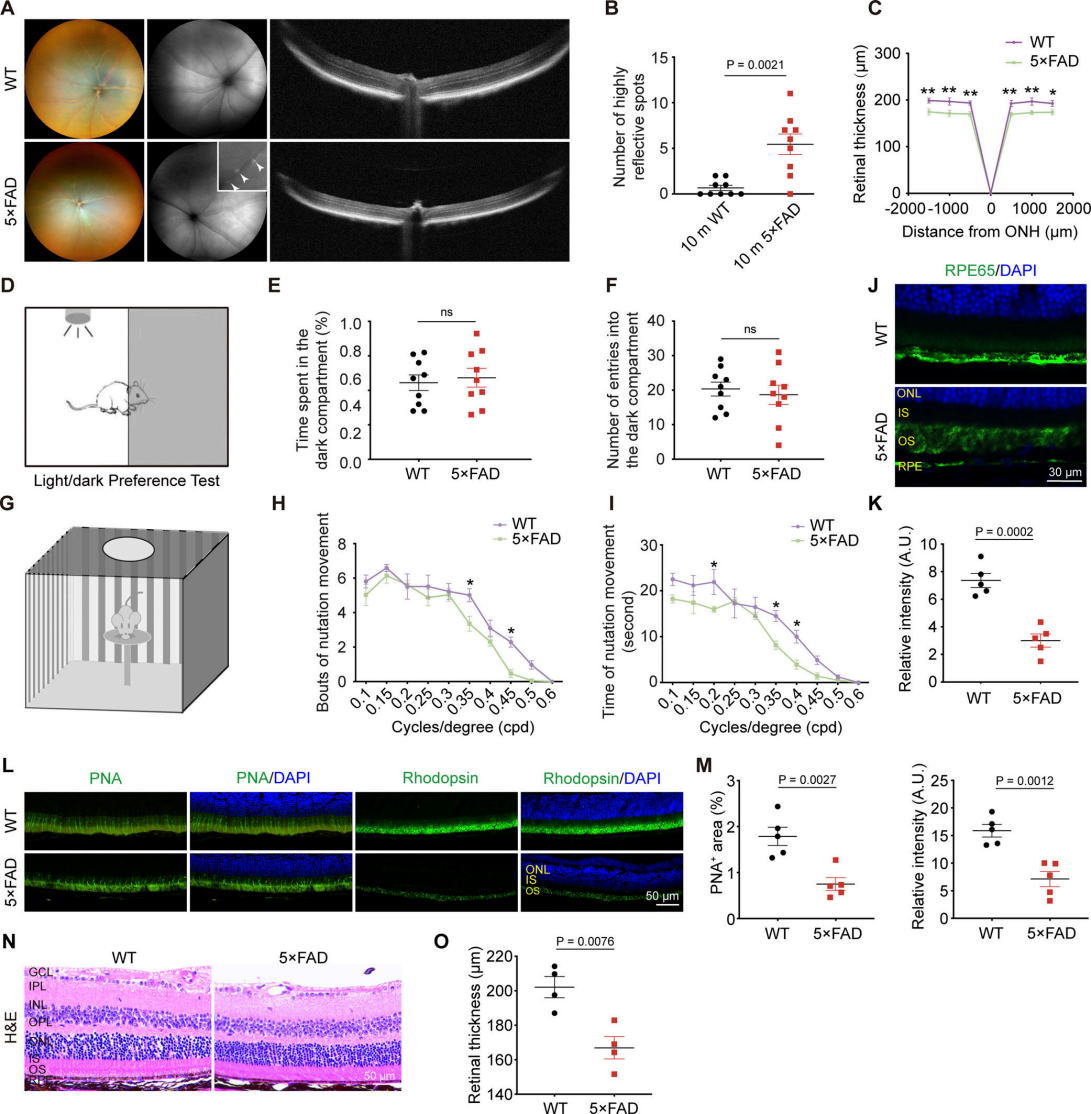
**Impairment of visual function and retinal pathology in 10-mo-old 5×FAD mice. (A)** Representative images of fundus, autofluorescence, and OCT in 5×FAD and WT mice, with white arrowheads indicating hyperautofluorescence spots in fundus images. **(B and C)** (B) Quantification of the number of hyperautofluorescence spots in the fundus and (C) retinal thickness measured by OCT (*n* = 9). **(D–F)** Schematic diagram of light–dark box experiments and quantification of the duration and number of entries into the dark box in WT and 5×FAD mice (*n* = 9). **(G)** Schematic diagram of optomotor response test in 5×FAD and WT mice (*n* = 9). **(H and I)** Quantification of (H) optomotor motion and (I) the duration of head movements for each grating density. **(J and K)** Representative images and quantitative analysis of RPE65 (green) staining in retinal cryosections from WT and 5×FAD mice (*n* = 5). **(L and M)** Immunofluorescence staining and quantification of PNA and Rhodopsin showing reduced expression levels in 5×FAD mice compared with WT mice (*n* = 5). **(N and O)** Representative images of retinal sections stained with HE and quantitative analysis of retinal thickness in WT and 5×FAD mice (*n* = 4). Representative of three independent experiments. All data are presented as mean ± SE of the mean (SEM). Statistical significance was assessed using the Mann–Whitney test (B, E, and F), two-tailed unpaired *t* tests (K, M, and O), or repeated-measures analysis of variance (ANOVA) followed by Bonferroni post hoc test (C, H, and I). *P < 0.05; **P < 0.01. GCL, ganglion cell layer; IPL, inner plexiform layer; INL, inner nuclear layer; OPL, outer plexiform layer; ONL, outer nuclear layer; IS, inner segment; OS, outer segment; RPE, retinal pigment epithelium.

5×FAD mice, a rodent model of AD, are well-described in the field of AD research (Oakley et al., 2006). We examined Aβ deposition in the ocular system of these mice with the 6E10 antibody. Consistent with Aβ deposition on the ocular system of AD patients, 6E10-positive Aβ plaques were found throughout the retina of 10-mo-old 5×FAD mice (Fig. 2, A and B) and predominantly associated with retinal vasculature (Fig. 2 C). In the optic nerve, besides the blood vessels, Aβ was found around the optic nerve sheath (Fig. 2, D and E). Furthermore, 3D reconstructed images showed that in 5×FAD mice, Aβ protein was deposited in the PVS of the retina (Fig. 2 F), Enzyme-linked immunosorbent assay (ELISA) revealed high levels of Aβ_1–40_ and Aβ_1–42_ in the retina of 5×FAD mice compared with age-matched wild-type (WT) mice (Fig. 2, G and H). Interestingly, Aβ was mainly present in the PVS along the arteries (Fig. 2, I–K). It was also distributed within the interstitial spaces in the retina and optic nerve. Aβ was also found in the optic nerve axonal compartment (Fig. 2 L), the optic nerve meningeal lymphatic vessels, and periorbital lymphatic vessels (Fig. 2, M and N). Additionally, in 10-mo-old 5×FAD mice, increased fundus autofluorescence spots (Fig. 3, A and B), impaired visual function (Fig. 3, D–I), abnormal RPE phagocytic function (Fig. 3, J and K), and retinal atrophy were observed (Fig. 3, L–O).

### Brain-derived Aβ is transported to the retina via a brain–eye glymphatic drainage route

**Figure 4.**
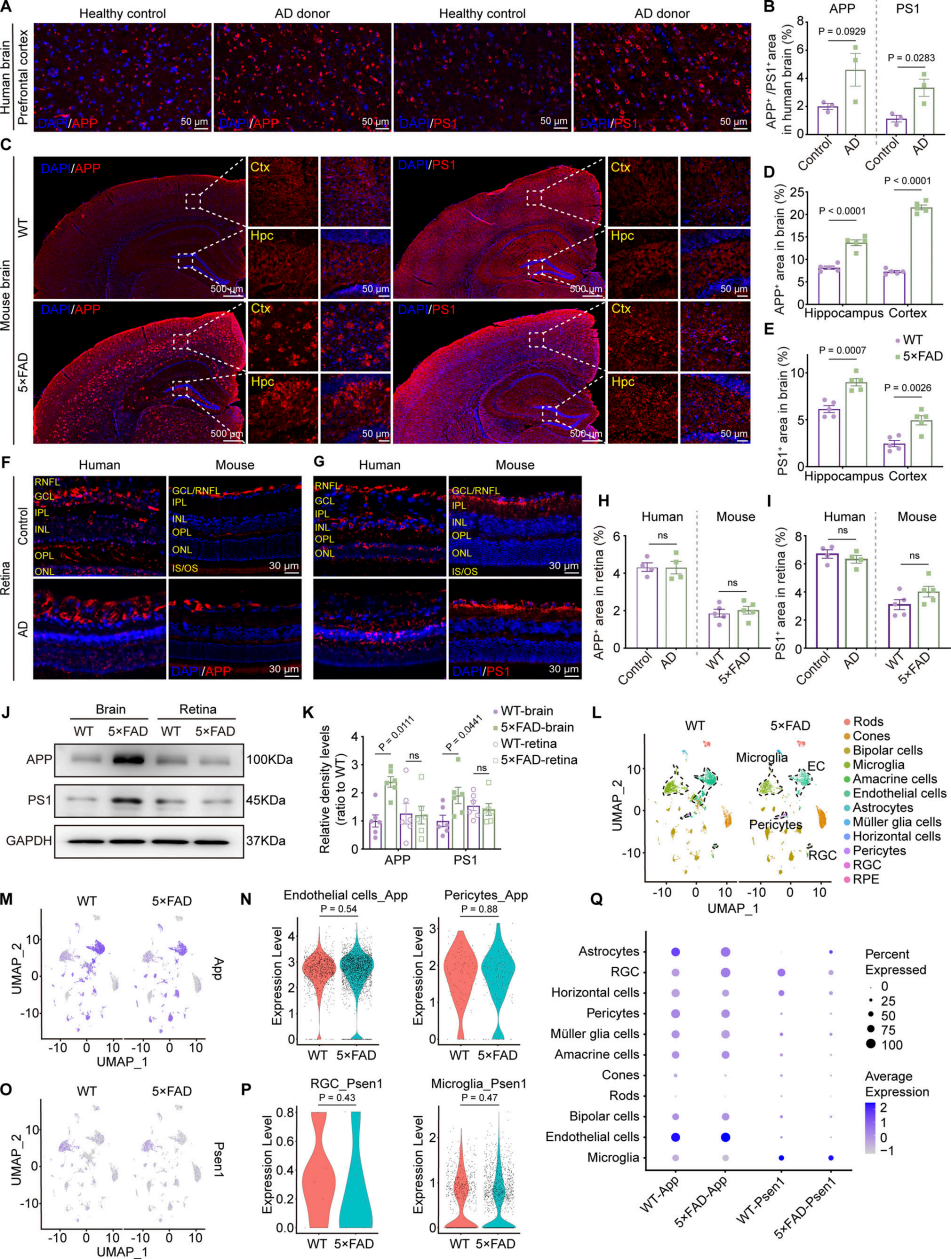
**Overexpression of APP and PS1 in the brain but not in the retina. (A, C, F, and G)** Representative immunofluorescence images showing the distribution of APP and PS1 in the brain (A and C) and retina (F and G) of AD donors and mice. **(B, D, E, H, and I)** Quantitative analysis revealed that, compared to healthy controls, the levels of APP and PS1 in the brains of AD patients show an increasing trend (*n* = 3). When compared to age-matched WT mice, the percentage of APP^+^ and PS1^+^ areas in the hippocampus and cortex increased in 5×FAD mice, but there was no significant difference observed in the retina (*n* = 5). **(J and K)** Western blot analysis of APP and PS1 protein levels in the brain and retina of WT and 5×FAD mice, with corresponding grayscale values (*n* = 6). **(L)** UMAPs showed cell clusters of retinal tissue in 10-mo-old WT and 5×FAD mice based on single-cell sequencing data. **(M)** UMAP clustering plot showing the distribution and expression levels of *App* in different cell clusters of retinas from WT and 5×FAD mice. **(N)** Violin plot showing the gene expression levels of *App* in retinal endothelial cells and pericytes from WT and 5×FAD mice. **(O)** UMAP clustering plot showing the distribution and expression levels of *Psen1* in different cell clusters of retinas from WT and 5×FAD mice. **(P)** Violin plot showing the gene expression levels of *Psen1* in retinal ganglion cells and macrophages/microglia from WT and 5×FAD mice. **(Q)** Gene expression dot plots showed the expression of *App* and *Psen1* in the retinas of WT and 5×FAD mice, and the results indicated no significant differences in RNA expression at the cellular level across various cell populations. Data are representative of two independent experiments. Data are presented as mean ± SEM. Statistical analysis was performed using two-tailed unpaired *t* tests (B, H, I, N, and P) or one-way ANOVA with post hoc Tukey tests (D, E, and K). Source data are available for this figure: SourceData F4.

Aβ is generated from amyloid β precursor protein (APP) through cleavages by β-secretase and γ-secretase. Presenilin 1 (PS1) protein is a critical component of γ-secretase. In the AD transgenic mouse model, 5×FAD mice express human *APP* and *PSEN1* transgenes with a total of five AD-linked mutations. Immunofluorescence staining revealed a significant increase in the expression levels of APP and PS1 in the brains of AD donors and 5×FAD mice (Fig. 4, A–E). However, the expression of APP and PS1 in the retinas of AD donors and mice showed no significant difference compared to the control group (Fig. 4, F–I). Western blot analysis further confirmed the same results at the protein level in both cerebral and ocular tissues (Fig. 4, J and K). These findings were further supported by the single-cell analysis from the retina of 10-mo-old 5×FAD and WT mice. The expression of *App* was primarily observed in endothelial cells, glial cells, pericytes, and bipolar cells, while *Psen1* was found in glial cells, bipolar cells, and ganglion cells. Interestingly, no statistical difference in the expression of *App* and *Psen1* in the respective cell types in the retinas was detected between 5×FAD mice and WT littermates (Fig. 4, L–Q). These data indicate that the elevated ocular accumulation of Aβ in 5×FAD mice and AD patients might not be due to increased local production.

**Figure 5.**
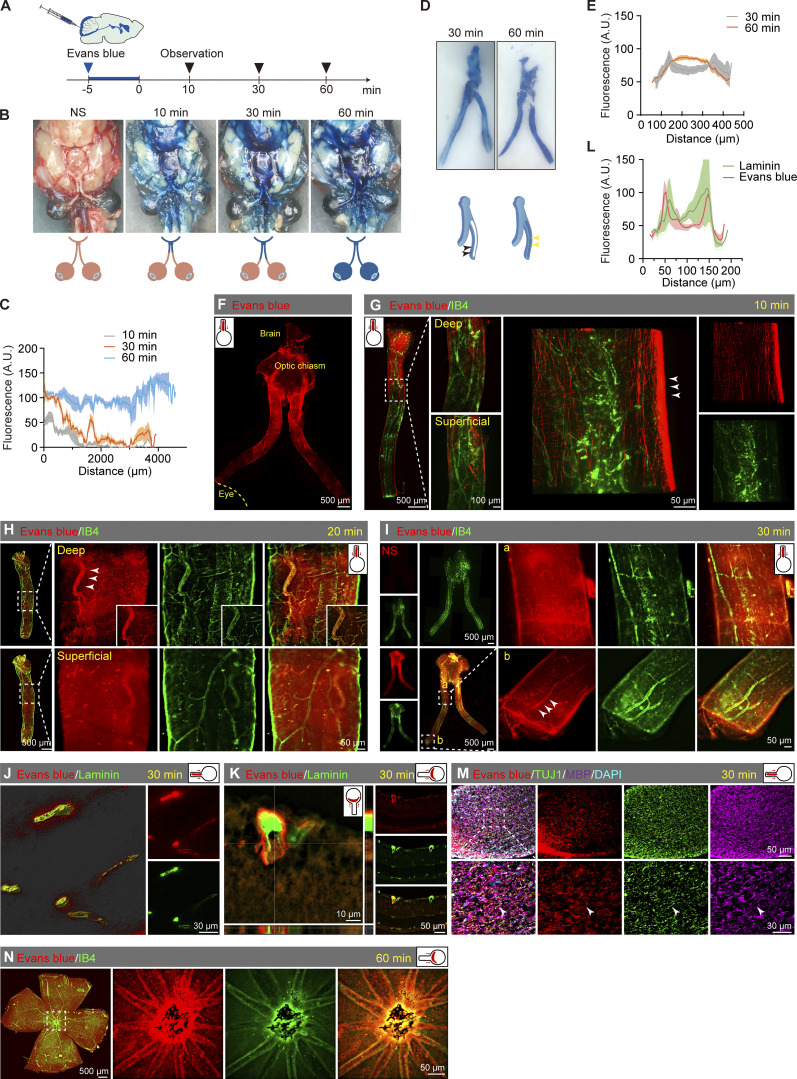
**The draining route for CSF tracers from the brain to the eye. (A)** Timeline of CM injection of Evans blue tracer. **(B and C)** Multitime point images of Evans blue spreading from the brain to the eye after injection and the corresponding statistical line chart of dye staining degree (*n* = 6). **(D and E)** 30-min and 60-min cross-sectional images of the optic nerve, along with a line graph representing the dye staining intensity along the yellow line. In the schematic diagram, the dye was visible at the sheath location at 30 min (black arrowhead), and at 60 min, the dye was within the optic nerve (yellow arrowhead). **(F)** Representative fluorescent microscopic image of Evans blue transportation along the optic nerve. **(G)** Image showing Evans blue transportation within the optic nerve sheath 10 min after injection, with the corresponding 3D image of IB4 staining (green) indicating blood vessels. **(H and I)** Representative images of Evans blue transportation within the optic nerve 20 and 30 min after injection along the blood vessels and nerve bundles. NS stands for the control group receiving brain injections of normal saline. **(J and K)** Representative images of Evans blue transportation within the optic nerve and retina 30 min after injection, with blood vessels stained by Laminin (green). **(L)** Line chart of fluorescence intensity showing the location relationship between Evans blue and blood vessels (*n* = 7). **(M)** Optic nerve sections demonstrated the distribution of Evans blue dye along myelinated axons along the optic nerve 30 min after injection. **(N)** Retinal flat mounts showing Evans blue transportation along retinal blood vessels 60 min after injection.

To test if it’s possible for CSF substrate to enter the neuroretina, we injected Evans blue into the cisterna magna (CM) of mice and observed their dynamic distribution in the optic nerve and retina at various time intervals (Fig. 5 A). Within 60 min after injection, the Evans blue color on the brain’s ventral surface increased over time and the color was darker along the optic nerve and optic tract. We observed an obvious time-dependent transport of the Evans blue tracer: the dye reached half of the optic nerve at 10 min, about two-thirds at 30 min, and the entire optic nerve by 60 min after injection (Fig. 5, B and C). When we longitudinally sectioned the optic nerve, we observed that the edge exhibited a heavier blue color 30 min after CM injection, while the interior showed a lighter color. However, after an additional 30 min of circulation, the dye was also found inside the optic nerve 60 min after CM injection (Fig. 5, D and E). Subsequently, the optic nerve was examined using a fluorescence microscope, revealing the occurrence of red fluorescence at the edge of the nerve and within the nerve along the vascular-like structures (Fig. 5 F). The detailed distribution of Evans blue was further examined at various time intervals by labeling blood vessels with isolectin B4 (IB4) or Laminin. At 10 min after CM injection of Evans blue, red fluorescence was observed surrounding the optic nerve sheath (Fig. 5 G). Later at 20 min after injection, Evans blue was found in the interior of the optic nerve along the PVS (Fig. 5 H). At 30 min, the fluorescence spread anteriorly along blood vessels close to the eyeball (Fig. 5 I). The examination of the optic nerve and retina cross-sections showed a clear association between Evans blue and blood vessels (Fig. 5, J–L). Also, at this point, the tracer was observed between the myelinated axonal compartment within the optic nerve (Fig. 5 M). At 60 min, the distribution of Evans blue extended throughout the retina along the blood vessels (Fig. 5 N).

**Figure S1.**
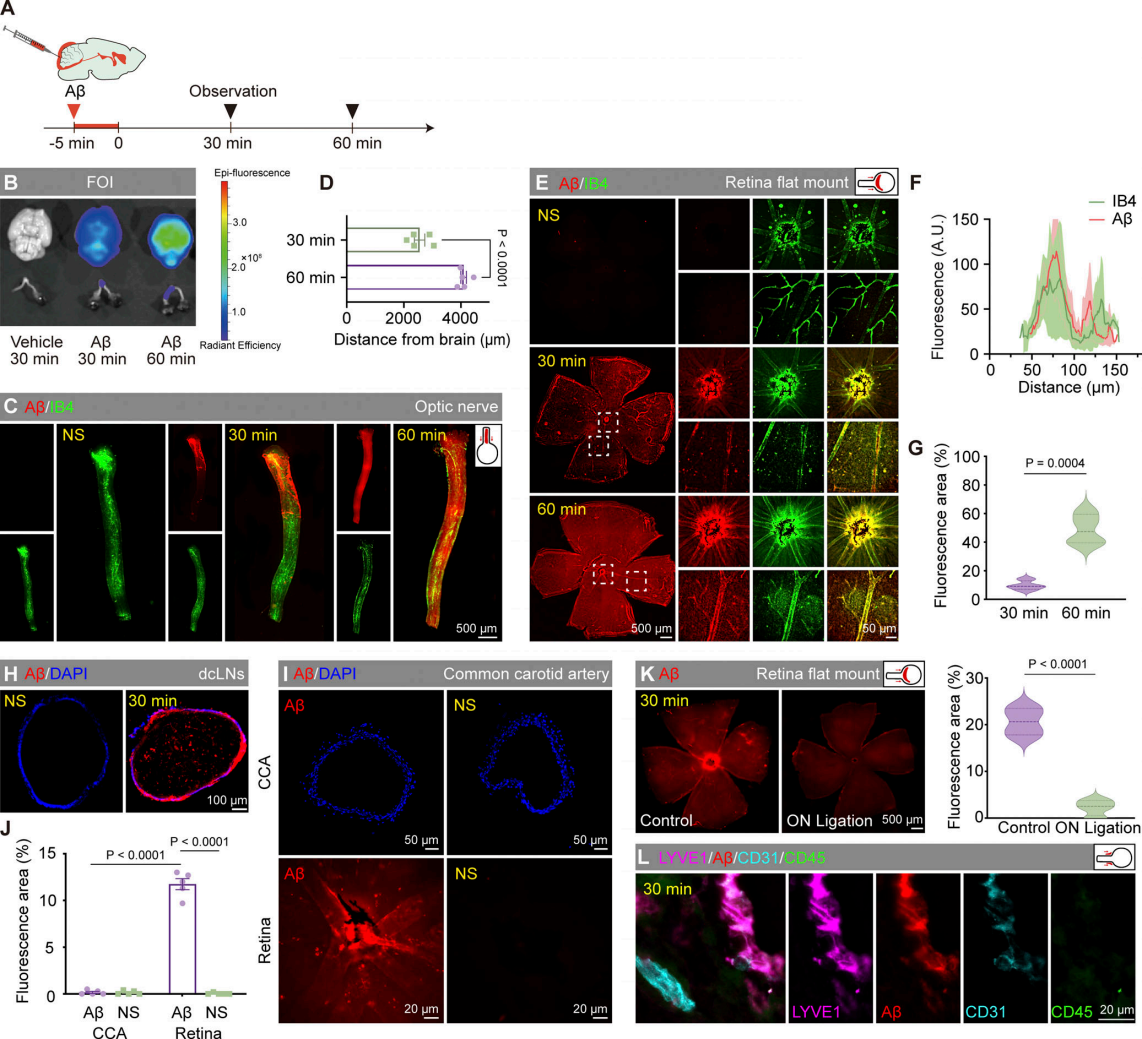
**Multitime point transport of Aβ in the optic nerve. (A)** Timeline of intracisternal injection of Aβ tracer. **(B)** Bioluminescence imaging showing the fluorescence intensity of Aβ in the brain and optic nerve 30 and 60 min after injection. **(C)** Representative images of Aβ distribution along the optic nerve in sections optic nerve after normal saline injection (30 min) and Aβ injection (30 min, 60 min). **(D)** Statistical graph of Aβ transportation distance in the optic nerve 30 and 60 min after injection (*n* = 5). **(E)** Representative images of short-range tracking of retinal Aβ transportation. **(F)** Fluorescence intensity profiles of the location of Aβ and IB4 in the vessels after a 30-min injection of CSF tracer (*n* = 8). **(G)** Statistical graphs of the percentage of area covered in short-range tracking experiments (*n* = 8). **(H)** Representative images showing the distribution of Aβ along the deep cervical lymph nodes (dcLNs) 30 min after saline injection and 30 min after Aβ injection. **(I and J)** Representative images and fluorescence intensity profiles of Aβ in the common carotid artery (CCA) and retina after normal saline injection (30 min) and Aβ injection (30 min) (*n* = 5). **(K)** Representative images and fluorescence intensity statistics showing the distribution of Aβ along the retina 30 min after Aβ injection following optic nerve ligation. **(L)** Immunofluorescence staining images of Aβ, Lyve1, CD31, and CD45 at the periorbital lymphatics, with CD45 labeling macrophages to exclude the possibility that Lyve1-labeled lymphatics are macrophages. Data are representative of three independent experiments. All data are presented as mean ± SEM. Statistical analysis was performed using two-tailed unpaired *t* tests (D, G, and K) or two-way ANOVA with post hoc Tukey tests (J).

**Figure 6.**
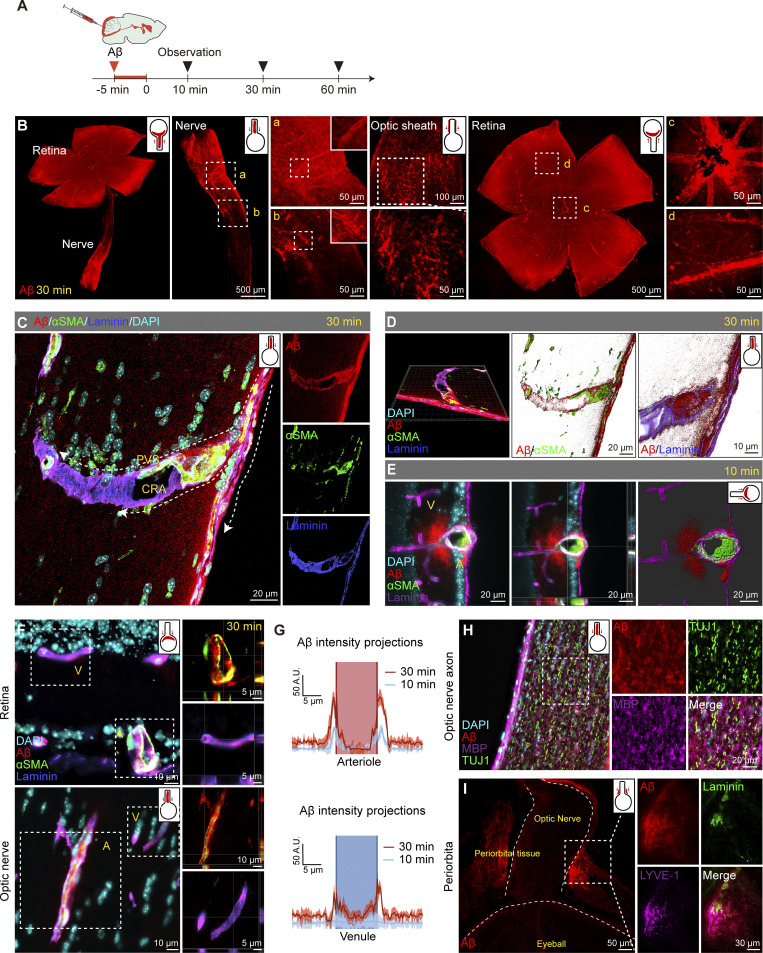
**The dynamic transportation of exogenous Aβ in the retina through the brain–eye drainage route. (A)** Short and long-term observation timelines of fluorescently labeled human Aβ injected into the CM. **(B)** Confocal images showing the transportation of Aβ in the retina, optic nerve, and its sheath after 30 min. **(C and D)** Immunofluorescence staining and 3D reconstructed images of the optic nerve revealed that Aβ travels along the PVS adjacent to the CRA, which was co-labeled with αSMA (green) and Laminin (blue). Arrowheads indicated the path of Aβ transport. **(E–G)** Immunostaining and statistical graphs demonstrated Aβ signals in arteries and veins of retina and optic nerve 10 and 30 min after CM injection (*n* = 6). Areas with αSMA^+^ Laminin^+^ staining were labeled as “A” for arteries, and areas with αSMA^−^ Laminin^+^ staining were labeled as “V” for veins. **(H and I)** Representative images of Aβ tracer signal 30 min after injection in the lymphatic vessels adjacent to the optic nerve, characterized by LYVE1^+^ Laminin^−^ staining, as well as in the gaps between myelinated axons within the optic nerve, marked by MBP and TUJ1.

The Aβ tracer followed a similar pattern entering the retina as used by Evans blue. The tracer was observed to occupy about half of the length of the optic nerve 30 min after injection, as shown by bioluminescence imaging (Fig. S1, A and B). At 60 min after injection, the tracer was further moved along the blood vessels in the optic nerve and retina (Fig. S1, C–G). The hAβ tracer was also observed in the deep cervical lymph nodes (Fig. S1 H). However, there was no distribution of hAβ in the common carotid artery at 30 min (Fig. S1, I and J). Additionally, we observed a significant blockade of the retinal signal following optic nerve ligation, indicating the hAβ transport depends on the optic nerve and is truly from the brain (Fig. S1 K). Further details of hAβ injected intracisternal distribution on the optic nerve and retina were revealed under a fluorescence microscope (Fig. 6, A and B). Initially, hAβ fluorescence was observed in the sheath and blood vessels of the optic nerve (Fig. 6, B-a and B-b), followed by its spread along the blood vessels from the central to the peripheral of the retina (Fig. 6, B-c and B-d). The fluorescence intensity of the optic nerve gradually decreased from the brain side to the eye side, indicating the directionality of hAβ movement (Fig. 6 B). 3D reconstructed images demonstrated the transport of hAβ along the optic nerve sheath, particularly in the subarachnoid space (SAS) between the arachnoid and pia mater. Additionally, higher magnification observations allowed for the elucidation of the details at the entry point where the tracer is transported along the PVS adjacent to the arteriole, infiltrating the optic nerve (Fig. 6, C and D). Interestingly, 10 min after CM injection, the hAβ tracer was primarily transported along periarterial space. In comparison, at 30 min, the tracer also appeared along the PVS of the deeper vascular network and concurrent signals of the hAβ tracer within the veins (Fig. 6, E–G). Furthermore, like endogenous Aβ in AD patients and 5×FAD mice, exogenous Aβ could also spread along the optic nerve axonal compartment (Fig. 6 H). We also found the transport of brain-derived hAβ in the optic nerve meningeal lymphatics and the periorbital lymphatics of 3-mo-old WT mice (Fig. 6 I and Fig. S1 L).

**Figure S2.**
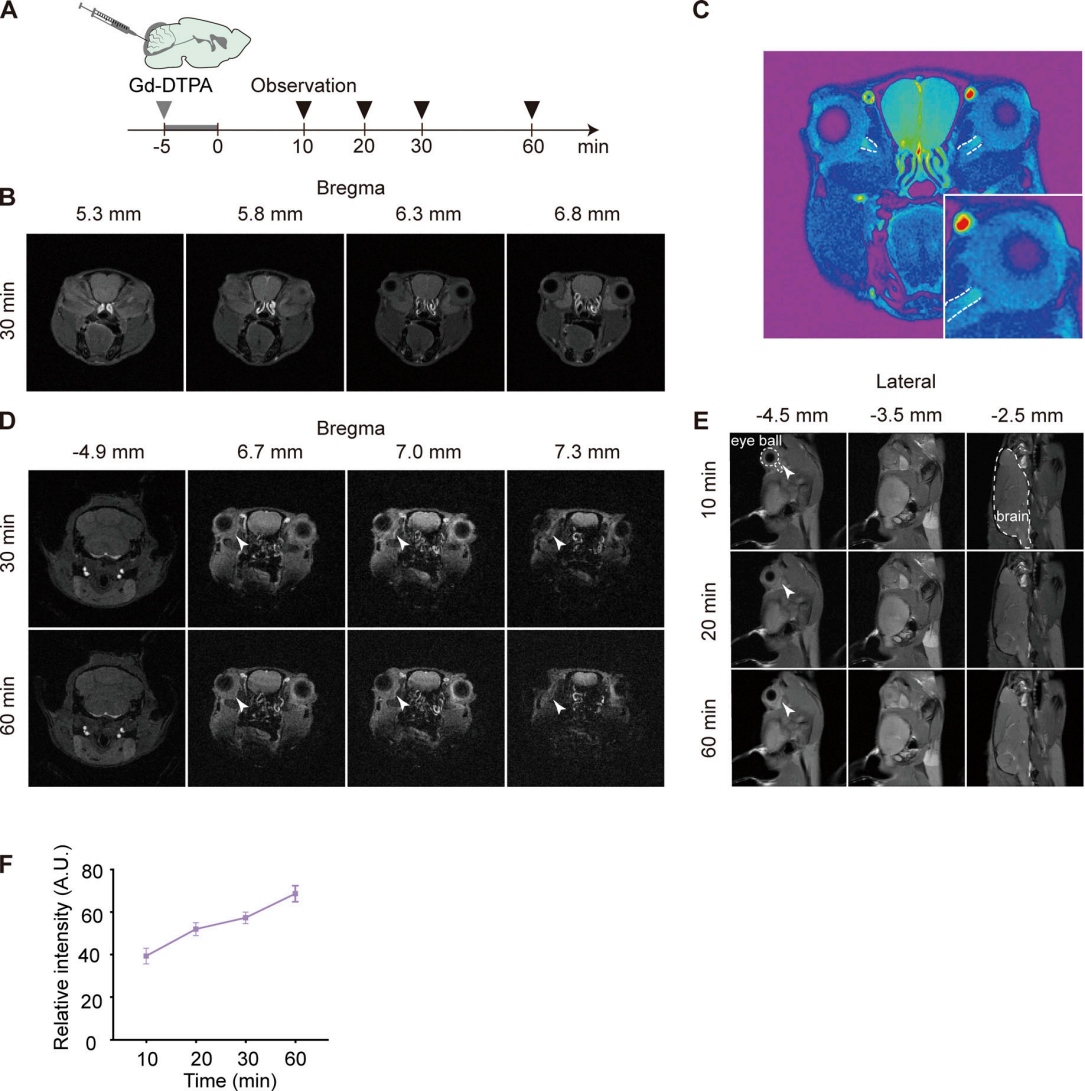
**Transport of the contrast agent along the optic nerve. (A)** Timeline of intracisternal injection of Gd-DTPA contrast agent. **(B)** MRI imaging of the brain after injection. **(C)** High-magnification image of the optic nerve in MRI. **(D and E)** Coronal and sagittal MRI images at different time points. A high reflectance signal of the contrast agent in the periorbital tissue was indicated by white arrowheads. **(F)** Line graph of contrast agent signal intensity in the eye area at different time points on MRI images.

Additionally, magnetic resonance imaging (MRI) imaging was employed, revealing the presence of gadopentetic acid (Gd-DTPA) in the optic nerve after CM injection that gradually intensified over an hour of imaging paradigm (Fig. S2, A–F). Together, through these tracking experiments that utilize different imaging techniques and tracers of varying properties, we identified a brain-to-eye transport pathway for the clearance of macromolecules, including toxic Aβ. Brain waste can be transported first along the optic nerve sheath and then reach the retina through two routes: via the PVS and the axons within the optic nerve. Additionally, the optic nerve meningeal lymphatics and periorbital lymphatics are both involved in removing brain-derived hAβ and possibly are downstream.

### AQP4 deficiency slows down the transport speed from the brain to the eye

Previous studies including the study from our own (Hablitz et al., 2020; Wang et al., 2022) have demonstrated that the glymphatic transport of toxic metabolites in the brain glymphatic system depends on AQP4 polarity. Structurally, there are similarities in the cellular and molecular architecture between the ocular glymphatic system and the brain glymphatic system (Wang et al., 2020). Thus, we postulated that AQP4 may mediate the transport of Aβ from the brain to the eye.

**Figure S3.**
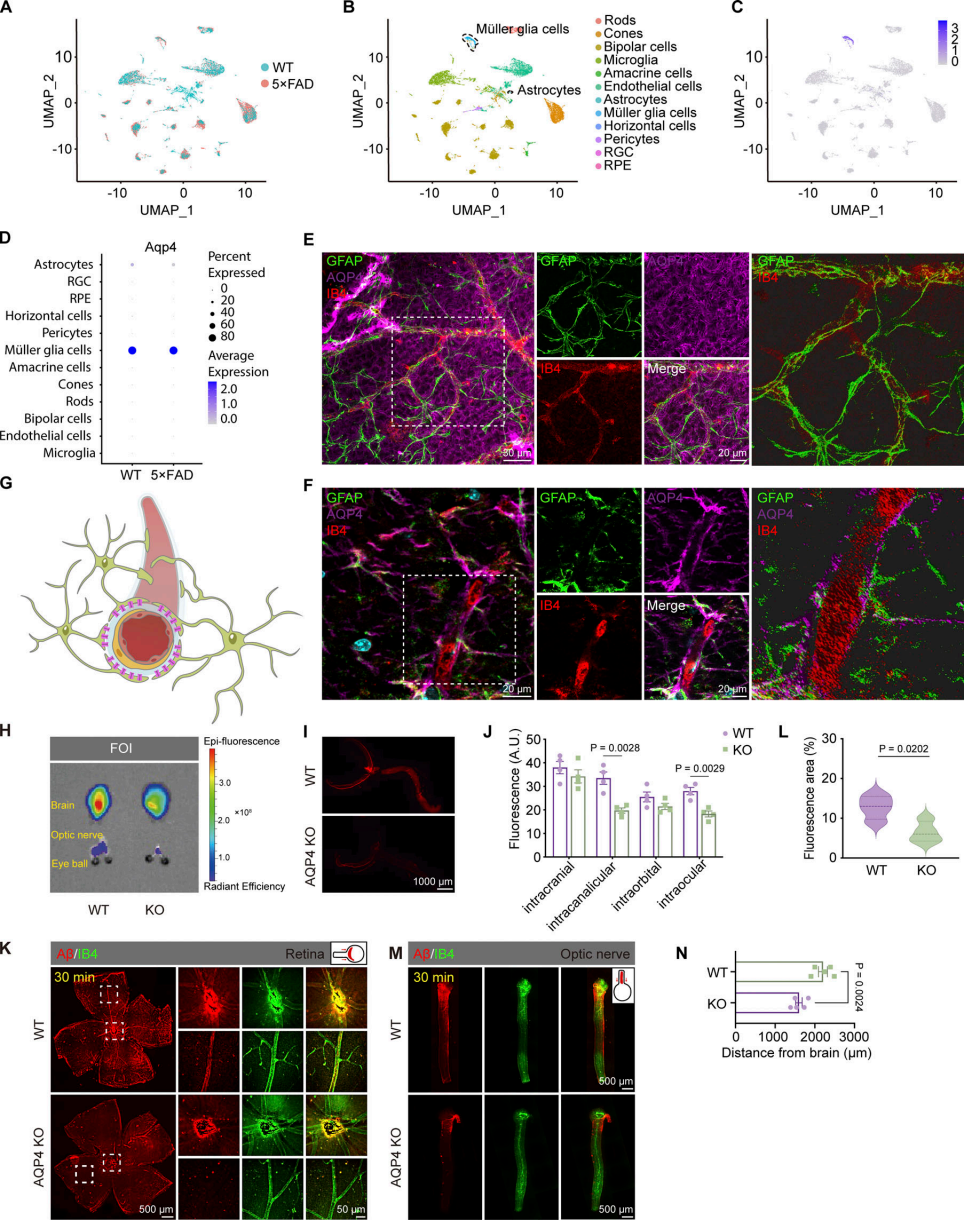
**Cellular distribution of AQP4 in the retina and optic nerve. (A)** UMAP cell type distribution of retinal cells in 10-mo-old WT and 5×FAD mice based on scRNA-seq data. **(B)** Identification of 12 cell groups by UMAP clustering. **(C)** Expression levels of *Aqp4* in the cell clustering plot. **(D)** The dot plot illustrated the percentage and expression levels of *Aqp4* across various cell populations. The data was obtained through single-cell analysis of the retinas from 5xFAD mice. **(E and F)** Representative immunofluorescence images of GFAP (green), AQP4 (purple), and IB4 (red) in the retina and optic nerve of 3-mo-old WT mice. **(G)** A schematic diagram depicting the relationship between astrocytes, AQP4, and vascular positioning, illustrating the localization of AQP4 on the endfeet of astrocytes, encompassing the blood vessels. **(H)** Bioluminescence imaging showing Aβ transportation fluorescence intensity in the brain and optic nerve of WT and AQP4 KO mice 30 min after injection. **(I and J)** Immunofluorescence staining of the optic nerve and eye sections of WT and AQP4 KO mice showing significantly reduced Aβ fluorescence intensity, with subregion statistical analysis in the intraocular, intraorbital, intracanalicular, and intracranial segments of the optic nerve (*n* = 4). **(K–N)** Representative confocal images and quantitative analysis showing the transportation of Aβ in the retinas and optic nerve of WT and AQP4 KO mice 30 min after injection (*n* = 8). Data are representative of two independent experiments. All data are presented as mean ± SEM. Statistical significance was evaluated using two-tailed unpaired *t* test (L and N), two-way ANOVA with post hoc Tukey test (J).

Our single-cell sequencing results of the retina from 10-mo-old WT and 5×FAD mice showed that *Aqp4* is primarily expressed by the retinal glial cells, including astrocytes and Müller glia cells (Fig. S3, A–D). We further performed immunofluorescent labeling of IB4, glial fibrillary acidic protein (GFAP), and AQP4 in the retina and optic nerve of 3-mo-old WT mice. We found that AQP4 exhibited a honeycomb-like pattern spreading across the entire retinal flat mount when observed from the outer nuclear layer, with a few areas of intense expression (Fig. S3 E). Additionally, AQP4 is primarily located around blood vessels labeled with IB4 in both the tissues of the retina and optic nerve (Fig. S3, E and F). This is consistent with previous reports indicating that AQP4 is also located at the endfeet of astrocytes (Nielsen et al., 1997).

Next, AQP4 knockout (KO) mice were employed to elucidate the precise involvement of AQP4 in the brain–eye transport of hAβ. The transportation distance of hAβ along the optic nerve was significantly shorter in AQP4 KO mice than in WT mice, as revealed by fluorescence microscopy and bioluminescence imaging (Fig. S3, H–J). Consistently, the retinal distribution area of hAβ in AQP4 KO mice was significantly less than that in WT mice (Fig. S3, K–N). Therefore, as hypothesized, the KO of AQP4 slowed down the transport speed of hAβ from the brain to the eye.

### Disrupted polarity or absence of AQP4 exacerbates retinal degeneration caused by brain-derived Aβ

To explore the long-term accumulation and clearance of brain-derived hAβ in the retina and the regulatory role of AQP4 in diseased states, we extended the time window of the hAβ CM injection experiment. We evaluated changes in visual function, retinal pathologies as well as AQP4 expression, and polarity states in the brain, optic nerve, and retina of AD patients and mice.

**Figure S4.**
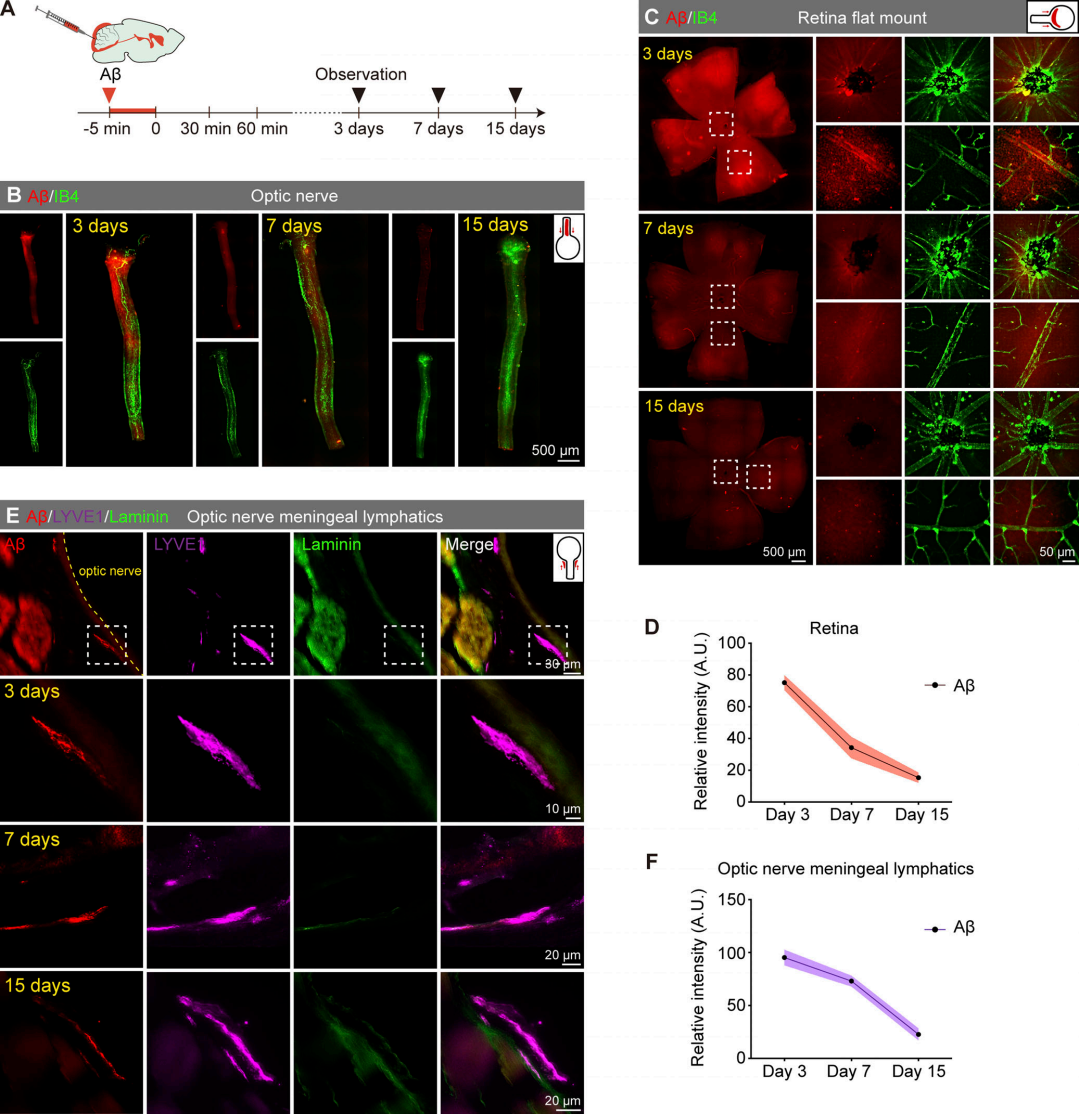
**Long-range tracing revealed the dissipation pattern of brain-derived Aβ in the retina. (A)** Long-term observation timelines of fluorescently labeled human Aβ injected into the CM. **(B and C)** Representative images of long-term tracking of the optic nerve and retinal Aβ transport. **(D)** Statistical graphs of the fluorescence intensity in long-term tracing experiments after CM injection (*n* = 8). **(E)** Representative images of long-term tracking of Aβ transport in optic nerve meningeal lymphatics. **(F)** Statistical graphs of the fluorescence intensity within lymphatics in long-term tracing experiments (*n* = 8). Data are representative of two independent experiments. All data are presented as mean ± SEM.

After injecting the hAβ tracer into the CM, we observed the prolonged dissipation of ocular hAβ at 3, 7, and 15 days in the WT mice. Strong hAβ labeling was still detectable in the optic nerve and retina 3 days after CM injection. However, the fluorescence intensity gradually decreased by the 7th and 15th days after CM injection (Fig. S4, A–D). We also observed the hAβ tracer signals excreted through ocular hAβ clearance pathways, including the periorbital lymphatics and optic nerve meningeal lymphatics at multiple time points. Notably, the hAβ signal within the lymphatic vessels was stronger at 3 and 7 days after CM injection and reduced at 15 days (Fig. S4, E and F). Combining previous research on the transportation of intraocular hAβ along the central retinal vein that eventually reaches the optic nerve meningeal lymphatics (Wang et al., 2020) and our results regarding the brain-to-eye transport of hAβ through the optic nerve sheath and optic nerve meningeal lymphatics, it is very likely that this perivenous space–optic nerve meningeal lymphatics pathway plays a critical role in the clearance of accumulated Aβ in the retina.

**Figure 7.**
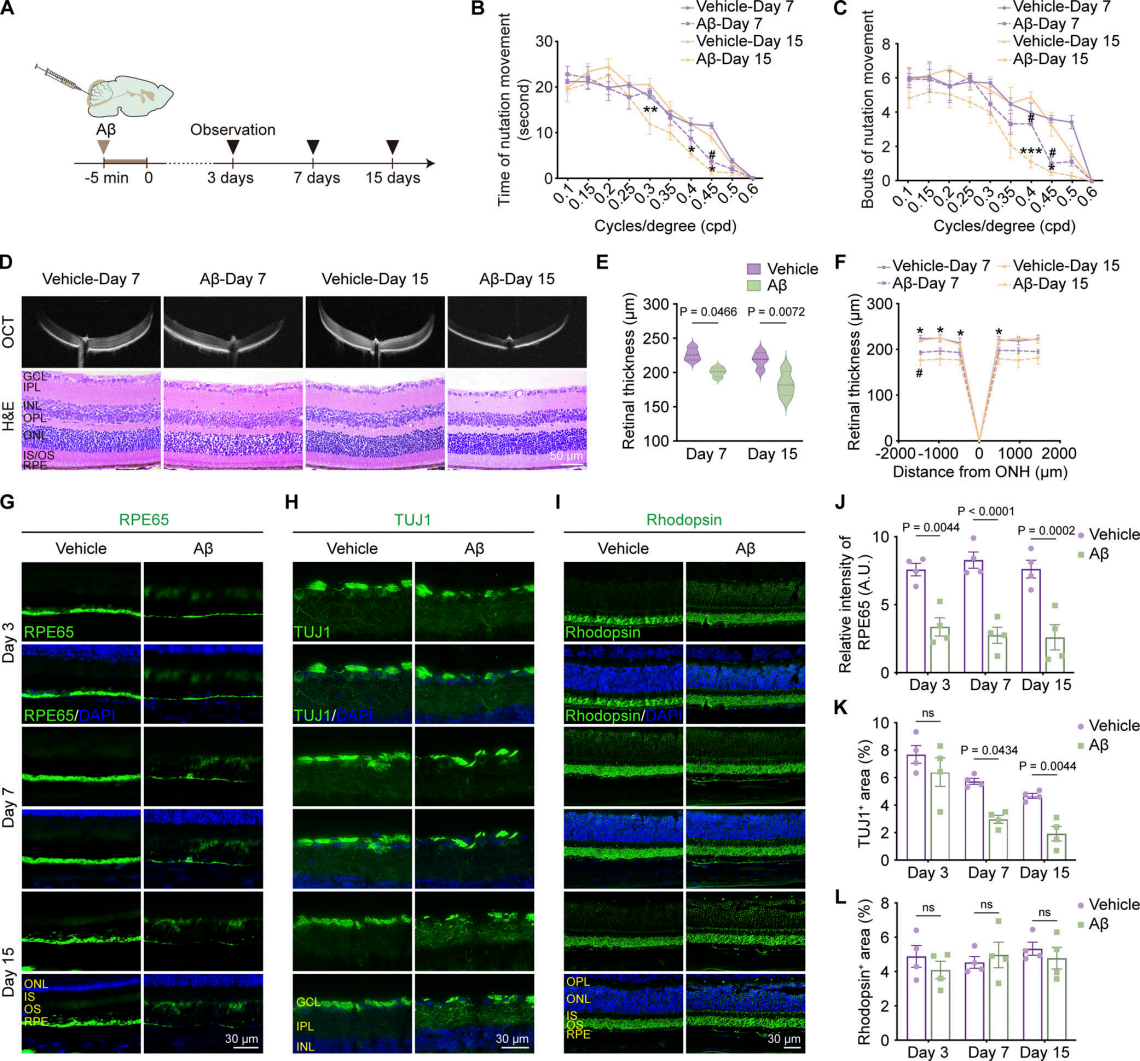
**Visual impairment induced by intracisternal injection of human Aβ**_**1–42**_**. (A)** Schematic of multi-time point CN injection of Aβ oligomers. **(B and C)** Optomotor response test showing the time and number of head motion responses under different grating densities in mice 3, 7, and 15 days after injection of hAβ tracer into the CM (*n* = 8). **(D–F)** OCT imaging and HE staining of the retina in mice at time points 7 and 15 days, with quantitative analysis of retinal thickness (*n* = 8). **(G and J)** Representative immunofluorescence staining images of RPE65 (RPE-specific marker) in retinal sections and corresponding quantitative analysis showing decreased RPE65^+^ fluorescence intensity after 3, 7, and 15 days after Aβ injection (*n* = 4). **(H and K)** Representative immunofluorescence staining images of TUJ1 in retinal sections and corresponding quantitative analysis showing decreased TUJ1^+^ area percentage at time points of 7 and 15 days (*n* = 4). **(I and L)** Representative immunofluorescence staining images of Rhodopsin in retinal sections and corresponding quantitative analysis showing no significant difference in Rhodopsin^+^ area percentage among the above three time points. Representative of three independent experiments. Data are presented as mean ± SEM. Statistical analysis was performed using one-way ANOVA with post hoc Tukey tests (E and J–L) or repeated measures ANOVA with Bonferroni post hoc tests (B, C, and F). *P < 0.05; **P < 0.01; ***P < 0.001, 15-day control group versus 15-day Aβ group; #P < 0.05, 7-day control group versus 7-day Aβ group.

**Figure S5.**
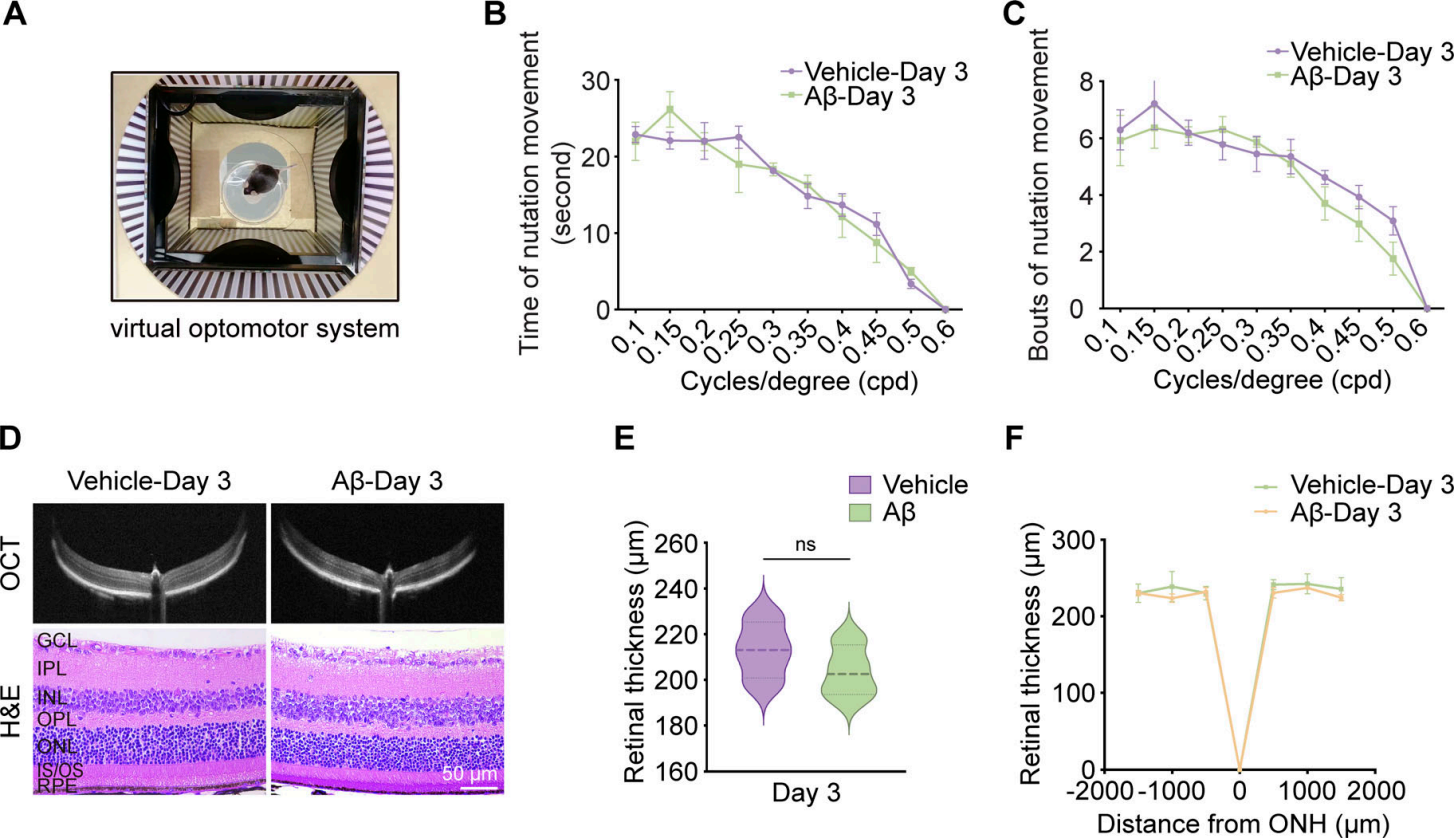
**No obvious visual impairments in mice after intracerebral injection of exogenous Aβ**_**1–42**_ **during 3 days. (A–C)** Actual photographs and quantitative analysis of optomotor responses at each grating density in mice 3 days after Aβ_1–42_ injection (*n* = 8). **(D–F)** OCT images of mice’s retinas 3 days after Aβ_1–42_ injection, with HE staining for retinal thickness measurement (*n* = 8). Data are representative of three independent experiments. All data are presented as mean ± SEM. Statistical significance was assessed using two-tailed unpaired *t* tests (E) or repeated measures ANOVA with Bonferroni post hoc tests (B, C, and F).

To assess the damaging impact of brain-derived Aβ on ocular visual function, we injected hAβ_1–42_ oligomers into the CM and monitored visual function in 3-mo-old WT mice at 3, 7, and 15 days after injection (Fig. 7 A). At the 3-day time point, no significant changes in vision were observed (Fig. S5, A–C), but visual impairment began to manifest at the 7-day time point and progressively worsened by the 15-day (Fig. 7, B and C). Consistent with the visual function results, optical coherence tomography (OCT) and hematoxylin and eosin (HE) staining analyses revealed no significant changes in retinal thickness at 3 days (Fig. S5, D–F), but a noticeable decrease was observed at 7 and 15 days (Fig. 7, D–F). The RPE65 staining was less intense 3 days after exogenous hAβ infusion, while β tubulin III (TUJ1)-labeled retinal ganglion cells exhibited abnormalities at 7 days. In contrast, Rhodopsin-labeled rod photoreceptor cells showed no significant changes at multiple time points (Fig. 7, G–L). These data demonstrate that brain-derived Aβ can directly induce retinal degeneration after being transported to the retina through the brain–eye glymphatic pathway. Together with the pathological findings from the mouse and human retina, it is likely that the brain-derived Aβ gradually accumulates in the retina and leads to retinal pathologies and visual impairments.

**Figure 8.**
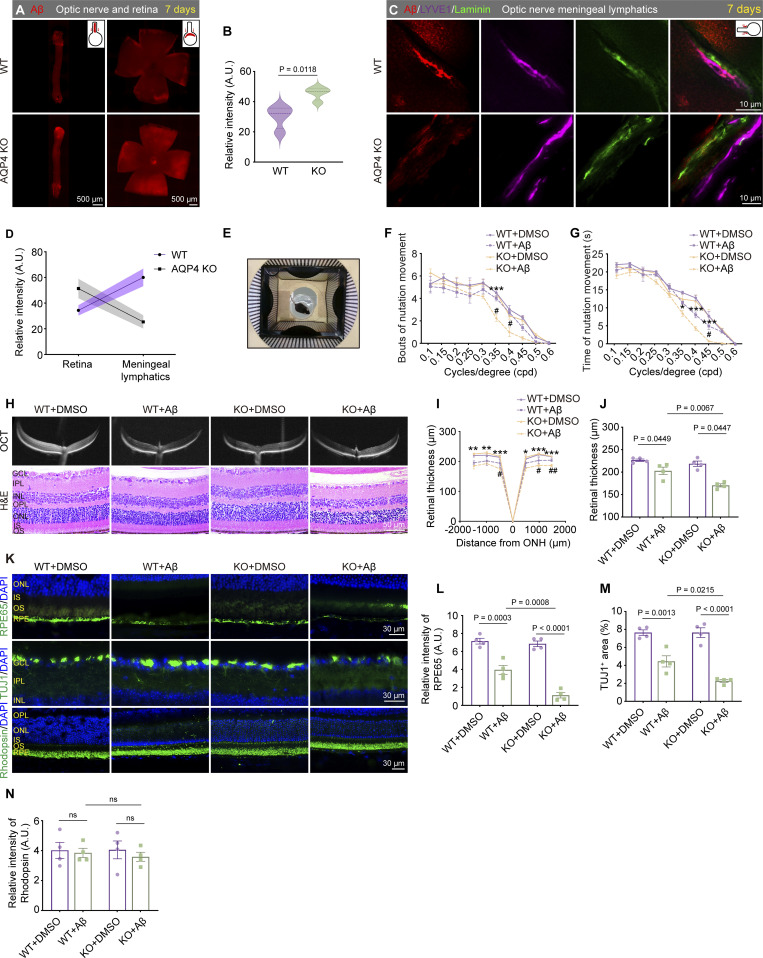
**AQP4 influences the brain-to-eye transport rate and exacerbates brain-derived Aβ-induced retinal damage. (A)** Representative confocal images showing the transport of Aβ in the optic nerves and retinas of WT and AQP4 KO mice 7 days after injection. **(B)** Quantitative analysis of the fluorescence intensity in the retinas 7 days after injection (*n* = 8). **(C)** Representative confocal images showing the transport of Aβ within optic nerve meningeal lymphatics of WT and AQP4 KO mice 7 days after injection. **(D)** Line chart of the fluorescence intensity in the retina lymphatics 7 days after injection (*n* = 8). **(E–G)** Optomotor response test in WT + DMSO group, WT + Aβ oligomer group, AQP4 KO + DMSO group, and KO + Aβ oligomer group (*n* = 10). Quantification of (F) optomotor motion and (G) the duration of head movements for each grating density. **(H–J)** OCT imaging and HE staining of the retina, with quantitative analysis of retinal thickness (*n* = 9). **(K–N)** Representative immunofluorescence staining images of RPE65 (L), TUJ1 (M), and Rhodopsin (N) in retinal sections and corresponding quantitative analysis (*n* = 4). Representative of three independent experiments. Data are presented as mean ± SEM. Statistical analysis was performed using two-tailed unpaired *t* tests (B) or two-way ANOVA with post hoc Tukey tests (J and L–N), repeated measures ANOVA with Bonferroni post hoc tests (F, G, and I). *P < 0.05; **P < 0.01; ***P < 0.001, AQP4 KO + DMSO group versus AQP4 KO + Aβ group; #P < 0.05, ##P < 0.01, WT+ Aβ group versus AQP4 KO + Aβ group.

Our previous results indicated that AQP4 KO mice could slow down the clearance of hAβ introduced by intravitreal injection through the perivascular pathway (Wang et al., 2020). The question remains whether the deletion of AQP4 would impact the retrograde ocular glymphatic clearance of hAβ introduced by intracisternal injection. The hAβ fluorescence intensity was higher in the optic nerve and retina of AQP4 KO mice than WT mice on the seventh day (Fig. 8, A and B), while the clearance of hAβ through the optic nerve meningeal lymphatic vessels was significantly reduced in AQP4 KO mice compared with WT mice (Fig. 8, C and D). The increased long-term accumulation of hAβ in AQP4 KO mice compared with the control mice may result from an inability of excretion, leading to the exacerbation of visual functional impairment (Fig. 8, E–G) and more severe pathological changes in the retina and RPE (Fig. 8, H–N). Therefore, AQP4 KO mice exhibit slowed transport from the brain to the eyes in the short term and decreased clearance of ocular Aβ in the long term.

**Figure 9.**
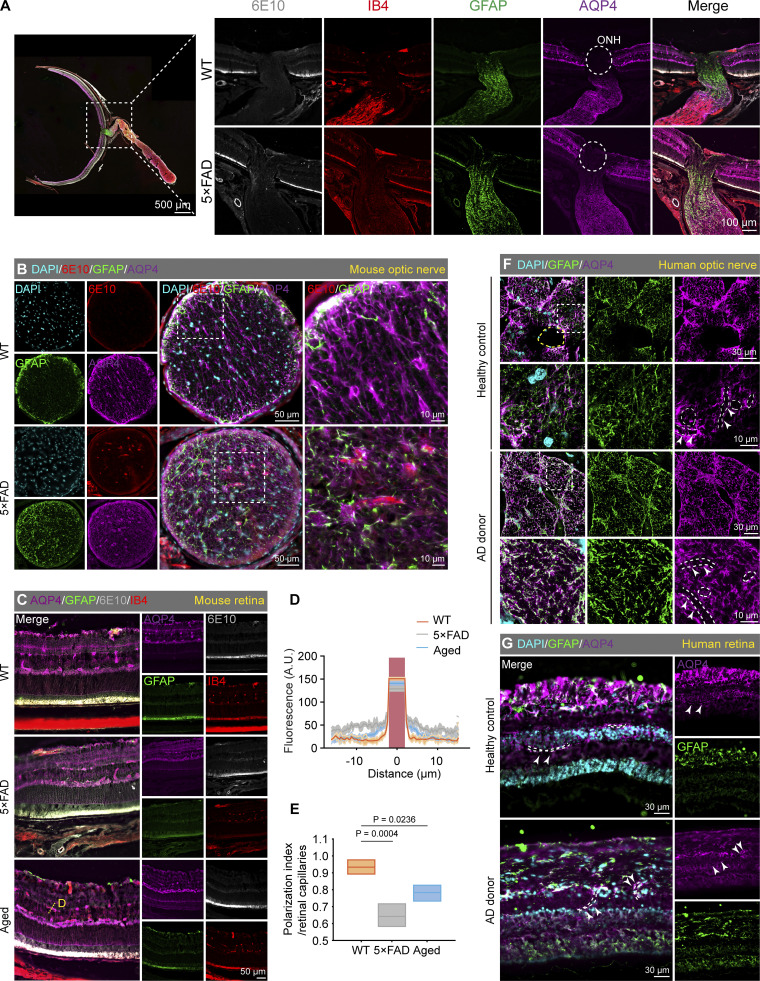
**Disruption of AQP4 polarity in the retina of AD patient, 5×FAD mice, and aged mice. (A)** Immunofluorescence images of the retina and optic nerve sections from 10-mo-old 5×FAD mice, showing staining of 6E10 (gray) for Aβ, IB4 (red) for blood vessels, GFAP (green) for astrocytes, and AQP4 (purple) for AQP4. The circular dashed outline indicated the location of the optic nerve head, where the expression of AQP4 was absent. **(B)** Representative images of immunofluorescence staining for 6E10, GFAP, and AQP4 in cross-sections of the optic nerve from WT and 5×FAD mice. **(C)** Representative immunofluorescence images of 6E10, GFAP, AQP4, and IB4 in the retina of WT, 5×FAD, and aged mice (18 mo). **(D)** Statistical analysis of AQP4 fluorescence intensity along blood vessels (yellow dashed lines), indicating more dispersed AQP4 localization in 5×FAD mice and aged mice in comparison to WT mice. **(E)** Box plot of the polarization index, where the polarization index was the peak fluorescence value of blood vessels minus the baseline fluorescence value, all values were normalized to the peak value (*n* = 6). **(F and G)** Representative immunofluorescence images of GFAP and AQP4 in the optic nerve and retina of AD donors and healthy controls. Dashed lines and arrowheads indicated the location of blood vessels (identified through AQP4 channel Z-axis imaging), revealing abnormal distribution of AQP4 in the optic nerve and retina of AD patients, indicative of polarity disruption. Representative of two independent experiments. All data are presented as mean ± SEM. Statistical significance was assessed using one-way ANOVA with Tukey’s post hoc test (E).

We further speculated that the accumulation of Aβ in both AD patients and 5×FAD mice may be associated with AQP4-mediated dysfunction in ocular Aβ clearance. The results showed that AQP4 signals adjacent to the retina vessels and optic nerve vessels of 5×FAD mice are lower than those in age-matched WT groups, while non-vascular areas exhibit stronger AQP4 signals (Fig. 9, A–E), suggesting a disruption of AQP4 polarity in the retina and optic nerve. Additionally, reduced AQP4 polarity was observed in AD patients’ retina and optic nerve tissues (Fig. 9, F and G). These changes in AQP4 may underlie the dysfunction of the ocular glymphatic system.

In summary, the absence of AQP4 or its disrupted polarity delays the influx rate of Aβ in the brain–eye pathway, hinders its long-term clearance from the retina, and impedes the drainage through the perivenous space-optic nerve meningeal lymphatics pathway. The obstruction of the clearance pathway in the ocular glymphatic system is a primary factor exacerbating retinal degeneration. This is a crucial factor contributing to the accumulation and pathology of retinal Aβ in AD patients and mice.

## Discussion

**Figure 10.**
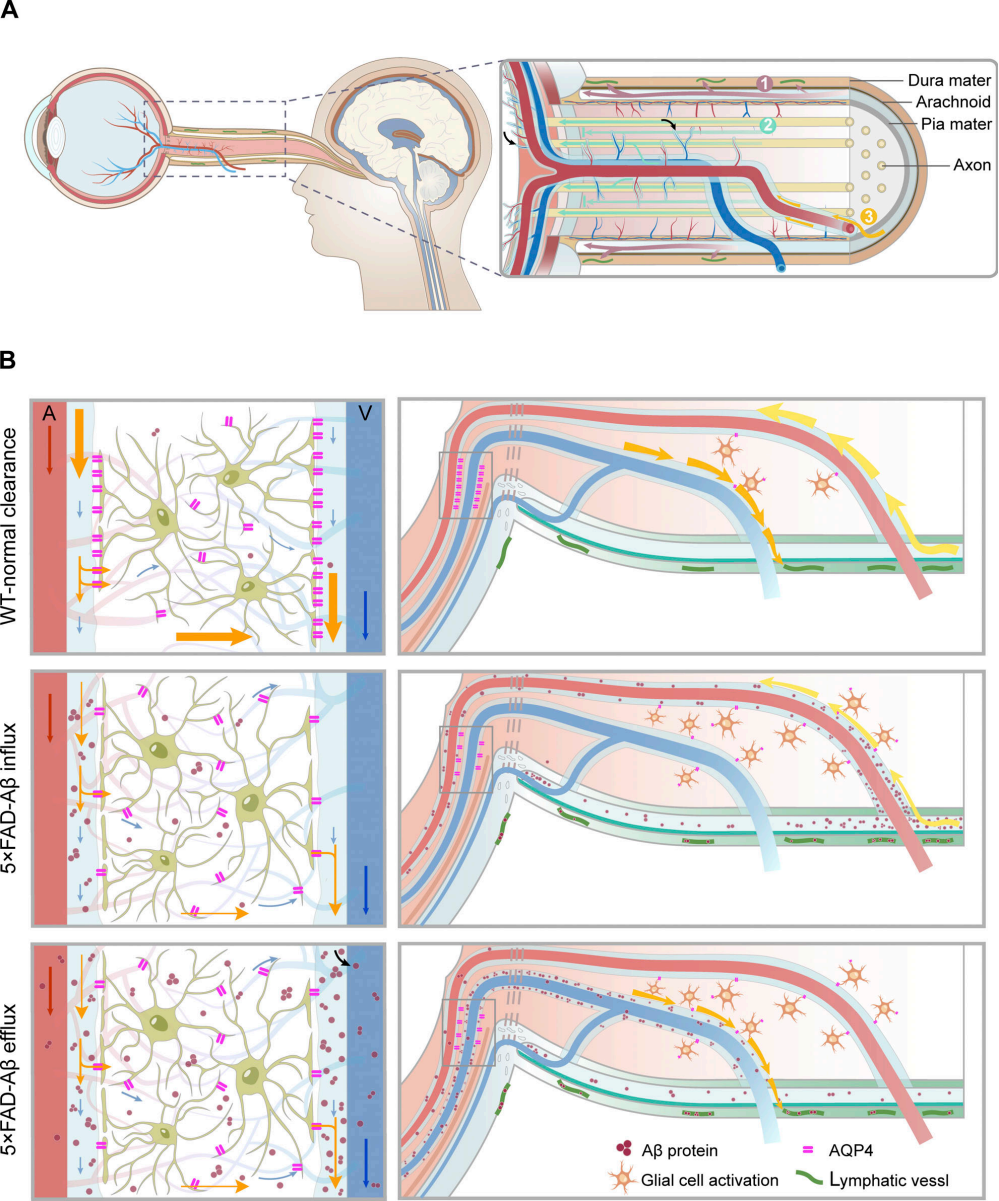
**The brain–eye transport pathway anatomy and AQP4-mediated glymphatic mechanism in AD-related retinal disorders. (A)** Anatomy of the brain–eye transport pathway. Brain–eye transport has three parts: transport along the SAS and optic nerve sheath, draining through optic nerve meningeal lymphatic vessels and periorbital lymphatic vessels. Second, transport along the myelinated axon spaces within the optic nerve toward the eye. Third, the transport of Aβ accumulated in the brain through the PVS of the CRA in the optic nerve, spreading to the retina. **(B)** Mechanism of AD-relevant retinal disease resulting from abnormal ocular glymphatic clearance by disrupted AQP4 polarity. In AD patients and 5×FAD mice, Aβ transported via the brain–eye pathway can enter the retina and accumulate through periarterial space in the short term. Over a longer duration, accumulated Aβ can be excreted through the perivenous space-optic nerve meningeal lymphatics pathway. During this process, the accumulated Aβ in the retina is transported from periarterial space to perivenous space and within the veins. Abnormal distribution of AQP4 in the PVS is a key factor causing abnormal accumulation of Aβ in the retina, subsequently triggering glial cell activation, inflammatory responses, retinal atrophy, and visual functional impairments.

Aβ accumulation in the ocular system and the consequent visual dysfunction were observed in AD patients and AD mouse models. In this study, we showed that the brain-to-eye transport route might be one of the main causes of AD-related ocular pathology. Aβ can be transported along the optic nerve via the SAS and the optic nerve sheath. Following that, Aβ enters the optic nerve and is transported along two routes that might be parallel to each other—the neural fibers and the PVS within the optic nerve—towards the retina, ultimately resulting in its deposition in the PVS adjacent to the retinal vessels (Fig. 10, A and B).

Transportation from the brain to the optic nerve is first described in rodents (Mathieu et al., 2017). More recently, a clinical report using computer tomography cisternography observed a noticeable decrease in contrast-loaded cerebrospinal fluid from the optic canal to the retrobulbar segment (Jacobsen et al., 2019; Xue et al., 2021), providing direct evidence for the existence of this pathway in humans.

Notably, a significant part of the brain-to-eye transport involves axons, similar to the transport by RGC axons from the eye to the brain (Wang et al., 2020). Here, we report hAβ tracer transport along the axons and compartments between axons from the brain to the eye. In the human, the RGC axons in the optic nerve, along with the central retinal artery (CRA), pass through the lamina cribrosa composed mainly of collagen and elastin fibers. Numerous axons converge to form a fascicle arranged in a polygonal pattern, with the fascicles surrounded by connective tissue septa. These septa connect to the peripheral pia mater and guide the vessels into the interior of the optic nerve (Salazar et al., 2019). In optic nerve samples from donors, we found a significant accumulation of Aβ in the axonal compartments of AD patients. In contrast, healthy controls showed only minimal amounts of soluble Aβ within the optic nerve axons. There is evidence showing that in the case of glaucoma, damage to the lamina cribrosa results in a shift of retinal Aβ outflow from intraxonal transport to extracellular transport (Wang et al., 2020). It will be interesting for future studies to explore the possibility of Aβ entering through a broken lamina into the retina in the AD setting.

Importantly, we observed that the Aβ tracer is transported along the PVS within the optic nerve and passes through the lamina cribrosa, continuing to transport along the retinal vessels and spread throughout the entire retina. A previous study described that intracisternal injection of CSF tracers with molecular weights of 10, 40, and 70 kDa could enter the optic nerve but not pass through the lamina cribrosa (Mathieu et al., 2017). In our study, using 0.96 kDa Evans blue and 5.36 kDa exogenous hAβ, we found that these small molecular weight tracers were transported from the brain to the retina. Additionally, injecting small amounts of tracer into the CM does not increase intracranial pressure (Iliff et al., 2013; Mathieu et al., 2013). Thus, the distribution pattern we report here represents a physiological condition, not an injection-induced artifact. We further clarified that the accumulation of Aβ in the PVS around the retinal and optic nerve arteries was higher than that around veins in AD mice. This might be due to the entry route of this brain-to-eye transport pathway being through the PVS of the arteries (Wang et al., 2020). Over time, Aβ is also present around veins, and some Aβ is absorbed through capillaries in the retina and optic nerve, entering the veins.

Here, using a murine model of AD, we revealed that the source of retinal Aβ is likely from the brain; we further investigated the clearance system of retinal Aβ. Aβ can be transported through periarterial influx–interstitial spaces-perivenous efflux, and finally, excreted through the perivenous–lymphatics pathway. We observed the presence of lymphatic vessels in both human and mouse samples at two locations. One is the periorbital lymphatic vessel located in the connective tissue adjacent to the sclera. Another one is the optic nerve meningeal lymphatic vessels situated in the optic nerve meninges, primarily in the dura mater (Wang et al., 2020; Yin et al., 2024). Interestingly, a previous study in hamsters showed that CSF from the terminus of the SAS at the orbital end can bypass the outer arachnoid layers of the optic nerve sheath, navigating through tortuous channels and microchannels to access the sclera and posterior periorbital connective tissue (Shen et al., 1985). CSF can also penetrate the intercellular space between arachnoidal cells and then be excreted through the optic nerve meningeal lymphatic vessels (Killer et al., 1999), ultimately drained into the deep cervical and submandibular lymph nodes (Ma et al., 2017). Recent research has revealed the eye–brain immunity between the posterior segment of the eye and the optic nerve meningeal lymphatics with the details remaining unknown (Yin et al., 2024). Based on these previous studies that could be linked to the pathway we report here, we argue that brain-derived Aβ, following reaching the SAS of the optic nerve, could pass from the terminus of the SAS via traverse tortuous channels and microchannels and ultimately excreted through the periorbital lymphatic vessels. Additionally, Aβ might also directly penetrate through the arachnoid, reach the meningeal lymphatic vessels, and be discharged into the peripheral lymphatic system. The optic nerve meningeal lymphatics and periorbital lymphatics may function similarly to the newly discovered fourth layer of the meningeal subdural lymphatic-like membrane (Mathieu et al., 2013; Mollgard et al., 2023; Tam et al., 2011). Furthermore, the periorbital lymphatic system not only serves as a downstream pathway of the ocular glymphatic system, draining ocular waste but also acts independently by directly uptaking CSF. Investigating whether the periorbital lymphatic system is impaired in AD, thus affecting the drainage of AD-related retinal waste to cervical lymph nodes, warrants further exploration. Regarding the molecular mechanisms of ocular lymphatic systems, we found that the abnormal polarity of AQP4 not only slows the entry rate, but, more importantly, hinders the self-excretion of the retina and the perivascular-lymphatics pathway. The combined effect of slow input and slow output ultimately leads to the accumulation of Aβ in the retina (Fig. 10, A and B).

The accumulation of Aβ in the retina can trigger visual functional impairment in AD. A growing body of evidence indicates that individuals with AD and mild cognitive impairment exhibit various eye impairments (Hart et al., 2016), such as abnormalities in visual sensitivity, contrast sensitivity, visual field, and motion perception, which may manifest before the onset of AD (Tzekov and Mullan, 2014). Consistently, pathological evidence confirmed the presence of Aβ deposits in the retinas of AD patients, primarily located in the innermost layers of the retina. Aβ distribution has been observed in the vascular lumen, endothelial cell surfaces, and within pericytes (Hart et al., 2016; Shi et al., 2020). Our study expands upon these findings by identifying the predominant accumulation of soluble Aβ in the blood vessels and periorbital lymphatic vessels throughout various ocular regions, including the retina, optic nerve, and choroid in AD patients. This deposition is distributed within the lumen of blood vessels, the middle layer of blood vessels, the PVS, and intercellular spaces. Similar Aβ deposition characteristics are also observed in 5×FAD mice (Kurochkin et al., 2018), and the accumulation of Aβ is attributed to a chronic and progressive imbalance between production and clearance (Shah et al., 2017; Zhou et al., 2020). In this study, the key enzymes responsible for Aβ production, namely APP and PS1, did not exhibit abnormal elevation in the retinas of 5×FAD mice and AD patients, suggesting that Aβ accumulation in the eyes could potentially be attributed to the brain-to-eye transport pathway. A previous study showed elevated levels of APP in the retinas of AD mice. However, it is important to note that the study utilized APP/PS1 mice, not 5×FAD mice (Mirzaei et al., 2019). This finding contrasts with the increased expression of APP and PS1 in aged brains (Kaja et al., 2015; Xu et al., 2019; Yoon et al., 2013). Likely, the inherent environment of retinas may not facilitate the upregulation of APP and PS1.

Collectively, Aβ in the brain can be transported to the eye, providing a significant source of ocular Aβ. The abnormality in the ocular glymphatic clearance system and the perivenous–lymphatics clearance pathway could lead to the long-term accumulation of brain-derived Aβ in the eyes, which might be the primary cause of Aβ abnormal accumulation. The presence of ocular Aβ may further lead to the activation of glial cells and ocular degeneration, resulting in visual impairment in AD patients. The polarity disorder of AQP4 in the retina and optic nerve leads to compromised substance exchange between the PVS and the interstitium, serving as a potential molecular pathway. Consequently, the proposed brain-to-eye transport emerges as an important pathogenetic pathway contributing to the development of AD retinopathy.

## Materials and methods

### Study design

The purposes of this study were as follows: (i) to investigate the ocular pathological changes in AD patients and mice and explore the mechanism of ocular Aβ deposition and visual impairment; (ii) to elucidate the existence of the brain-to-eye clearance pathway and the ocular Aβ deposition in AD mice resulting from impairment of the ocular glymphatic clearance system; and (iii) to explore the molecular mechanism of the ocular glymphatic clearance system. We used 5×FAD mice to simulate the ocular condition of AD. Subsequently, we conducted a comprehensive assessment of visual function, examined retinal and RPE pathology, and performed single-cell sequencing of the 5×FAD mouse retina to analyze the expression of related genes. Additionally, we explored the brain-to-eye pathway through cerebrospinal fluid tracing experiments, mouse MRI, and bioluminescence imaging. We also investigated the molecular mechanisms underlying the regulation of the ocular glymphatic clearance system in 5×FAD mice and AQP4 KO mice. The Animal Management and Use Committee of Nanjing Medical University (IACUC-1812054) granted approval for all experiments conducted in this study. Animals were allocated randomly to various treatment groups. The sample size was determined based on previous experience to ensure sufficient statistical power. The eye donors (one age 59, the other age 90) and brain donors (three are AD, and three are healthy controls) were sourced from the National Health and Disease Brain Tissue Resource Library, Nanjing Medical University Subcenter. Prior to their demise, informed consent was obtained from the donors, and the study received approval from the Medical Ethics Committee of Nanjing Medical University (no. 2018-633). The number of experimental repetitions was indicated in the figure legends.

### Experimental animals

WT mice (C57BL/6J background) and B6.Cg-Tg (APPSwFlLon, PSEN1*M146L*L286V) 6799Vas/Mmjax (5×FAD, JAX 008730) were purchased from the Jackson Laboratory. AQP4 KO mice were generated previously (Fan et al., 2005). All mice strains were housed in a specific pathogen-free environment at Nanjing Medical University Animal Center which was maintained under suitable temperature and humidity conditions, subjected to a 12-h light/12-h dark cycle, and provided with standard rodent food and autoclaved tap water.

### Ophthalmic examination

In the visual light–dark box experiment, a box with a small door was utilized, divided into equal halves of dark and light areas. During the experiment, the mice were allowed to move freely between the two compartments for 8 min. The camera recorded the duration and frequency of their presence in each compartment to evaluate their light-dependent visual behavior. Additionally, for the optomotor response experiment, a device with four display screens arranged in a square configuration was utilized. The mice were allowed to move freely on a centrally fixed platform, while the screens exhibited dynamic black and white gratings. A camera recorded the tracking response time and frequency of the mice for a total of 12 min, divided into 10–12 stages with different grating densities. At each stage, the grating rotated clockwise for 30 s, followed by a counterclockwise rotation for 30 s, with a 10-s pause in between. The grating densities for different stages were sequentially set as 0.10, 0.15, 0.20, 0.25, 0.30, 0.35, 0.40, 0.45, 0.50, and 0.60 cycles per degree, measuring the number of grating cycles within a 1° visual field. By recording the optomotor response time and frequency of the mouse’s head movement in response to the rotating visual stimulus, a statistical analysis was performed to evaluate the visual acuity of the mice. To conduct fundus photography, OCT, and autofluorescence imaging, the mice were anesthetized and their pupils dilated. Fundus photographs were captured using the CLARUS500 retinal camera (Carl Zeiss), fundus autofluorescence was observed using the Heidelberg Retina Angiograph 2 (HRA-2, Heidelberg, Germany), and OCT images were obtained using the CIRRUS HD-OCT500 (Carl Zeiss) to measure retinal thickness.

### CM injection

The mice were anesthetized by intraperitoneal injection of ketamine and xylazine. After anesthesia, they were positioned in a stereotaxic apparatus in a prone position. Following disinfection of the skin, an incision was made and muscles were gently separated to expose the dura mater outside the foramen magnum. Using a Hamilton microsyringe, 5 μl of Evans blue solution (10 mg/ml, catalog: E2129; Sigma-Aldrich), human fluorescently labeled Aβ_1–42_ (0.05 μg/μl, catalog: AS-60480-01; AnaSpec), or human Aβ_1–42_ oligomers (100 μmol/liter, catalog: 107761-42-2; NJPeptide) were slowly injected into the CM at a rate of 1 μl/min. The Evans blue solution was prepared by dissolving Evans blue powder in physiological saline to make a 10 mg/ml solution. The fluorescently labeled human Aβ_1–42_ was dissolved in physiological saline to make a 0.05 μg/μl solution. The human Aβ_1–42_ oligomers were first dissolved in DMSO to make a 500 μmol/liter solution and then diluted in physiological saline to make a 100 μmol/l solution. After injection, the needle was left in place for 5 min to prevent leakage and then slowly withdrawn. The incision was sutured, and the mice were placed on a warm blanket to recover from anesthesia. The control group was injected with an equivalent amount of solvent using the same procedure.

### Tissue preparation

After euthanasia, the eyes and brains were removed. To obtain frozen sections of the eyes, the eyeballs were immersed in FAS eye fixation solution (G1109; Servicebio) for 30 min and then fixed in 4% paraformaldehyde (PFA) at 4°C overnight. The fixed eyeballs, after removal of the anterior segment, underwent dehydration in sucrose solutions of varying concentrations (10%, 20%, and 30%) and were subsequently embedded in the OCT compound (Sakura Finetek). The resulting embedded samples were sliced into sections measuring 8 or 12 μm in thickness using a cryostat (CM1950; Leica). To prepare retinal and optic nerve flat mounts, eyeballs were initially fixed in 4% PFA at 4°C for 2 h. After removing the anterior segment, the retina and optic nerve were dissected. For frozen brain sections, brain tissues were first fixed in 4% PFA at 4°C overnight and then dehydrated in a 30% sucrose solution before being embedded in OCT. The frozen brain tissues were sliced into coronal sections measuring 30 μm in thickness. In preparing human eye tissue, eyeballs were fixed in a 4% PFA solution at 4°C overnight. Subsequently, under the observation of a microscope, eyeballs were dissected and the dissected tissues were dehydrated in a 30% sucrose solution. The tissues were then embedded in OCT before being cut into sections with a thickness of 12 μm using a cryostat. Additionally, paraffin-embedded human prefrontal cortex tissues were cut into sections with a thickness of 5 μm using a paraffin slicing machine (RM2135; Leica).

### Immunofluorescence staining

For retinal and brain cryosections, the sections were permeabilized with a blocking solution containing 0.3% Triton X-100 and 5% bovine serum albumin at room temperature for 1 h. Following this, the sections were incubated with primary antibodies (see Table S2) at 4°C overnight. After being washed with PBS three times, the sections were incubated with secondary antibodies (see Table S2) at room temperature for 2 h. DAPI (4′,6-diamidino-2-phenylindole, 1 μg/ml) was used to label the cell nuclei for 5 min. For human eye sections, Sudan Black B staining was performed for 5 min to remove any background interference. Subsequently, the stained samples were mounted and observed using a STELLARIS STED confocal microscope (Leica), a fluorescence microscope (DM4000B; Leica), and an inverted fluorescence microscope equipped with a thunder imaging system (DMi8; Leica).

### Western blot

Retina and brain tissues were homogenized in radioimmunoprecipitation assay (RIPA) lysis buffer and centrifuged at 12,000 rpm for 15 min at 4°C. The resulting supernatant was mixed with the loading buffer and boiled at 95°C in a metal bath for 10 min. The protein samples were then loaded into designated sample wells on polyacrylamide gels ranging from 10 to 15% and subjected to electrophoresis for separation. The separated proteins were transferred onto Polyvinylidene difluoride (PVDF) membranes. Following transfer, the membranes were blocked in a solution of 5% skim milk prepared in TBST (pH 7.5, 10 mM Tris-HCl, 150 mM NaCl, and 0.1% Tween 20) at room temperature for 1 h. Subsequently, the membranes were incubated with primary antibodies (Table S2) overnight at 4°C. On the following day, the membranes underwent three washes with TBST for 10 min each, followed by incubation with HRP-conjugated secondary antibodies (Table S2) at room temperature for 1 h. Subsequently, the membranes were washed five times with TBST for 10 min each, and chemiluminescence detection was performed using an imaging system (ImageQuantLAS4000mini, version 1.2). The grayscale values of the bands were then analyzed using ImageJ software (RRID: SCR_003070).

### Thioflavin-S staining

All samples were stained with 1% Thioflavin-S (Cat#1326-12-1; Sigma-Aldrich) for 5 min, followed by a PBS wash and subsequent differentiation in 70% alcohol for 1 min. After three additional PBS washes, the samples were affixed onto glass slides and visualized using both a fluorescence microscope (DM4000B; Leica) and an inverted fluorescence microscope equipped with a thunder imaging system (DMi8; Leica).

### HE staining

For paraffin sections of the retina, the sections underwent dewaxing with xylene and gradient ethanol, followed by staining with HE. After mounting, the retinal tissue structure was observed using a bright-field microscope (DM4000B; Leica), and the thickness of each retinal layer was analyzed using ImageJ software.

### Image processing

The ImageJ software was used for image processing and analysis. The grayscale threshold analysis method was used to quantify the area of positive signal with fixed values assigned to the minimum and maximum grayscale levels. The ratio of the positive signal area to the total area was then calculated to determine the percentage of positive signal area. For morphological analysis of microglia, Sholl analysis was performed using ImageJ for each cell (Green et al., 2022). The cell body was designated as the central point, starting from a radius of 5.5 μm and increasing by 2 μm for each subsequent circle. The intersections between microglial branches and different circles were quantified and a Sholl plot was generated. Each cell was subjected to quantitative analysis of branch length and number of branches. Additional parameters, including the occupied area and cell body area, were analyzed using ImageJ software. For the analysis of AQP4 polarity, the ImageJ software was used to perform a statistical analysis on the mean AQP4 immunofluorescence intensity in 30-μm segments surrounding blood vessels (identified by AQP4 localization around them). Additionally, 100 μm segments extending from the meninges to the cortex were assessed to quantify AQP4 polarity by comparing the expression ratio of AQP4 in the perivascular and parenchymal domains (Kress et al., 2014; Zhang et al., 2020; Wang et al., 2022).

### ELISA

Retina was homogenized in RIPA lysis buffer with protease inhibitors and centrifuged at 12,000 rpm for 15 min at 4°C. Supernatants were set aside for measurements of the concentrations Aβ_1–40_ and Aβ_1–42_. Their expression was measured using ELISA kits (cat# ZC-37880; cat# ZC-37881; Zci Bio) as per the manufacturer’s protocol.

### Flow cytometry (FACS)

After the euthanasia of mice, the retinas were isolated in a high-glucose DMEM medium containing 2% FBS. Subsequently, they were transferred to a digestion solution containing 2.5% papain and 1 μg/ml DNase and digested at 37°C for 5 min. Next, the digestion solution was removed and the cell suspension was pipetted up and down until the absence of visible tissue fragments, followed by filtration through a 40-μm mesh (Cat#352351; BD Falcon) to obtain a single-cell suspension. Subsequently, centrifugation at 300 *g* for 5 min was performed, leading to the removal of the supernatant and resuspension of the cells in a high glucose DMEM medium containing 2% FBS. CD31 and CD45 were used as positive screening markers, while CD73 was served as a negative screening marker. The cells were then incubated at 4°C in the dark for 30 min. Following the washing procedure with high glucose DMEM medium containing 2% FBS, a limited number of unlabeled cells were stained with Trypan blue for cell viability detection, and then CD31^+^, CD45^+^, and CD73^−^ cells were sorted using the FACS ARIA II SORP flow cytometer (BD Bioscience).

### Single-cell RNA sequencing (scRNA-seq)

The cells were processed into barcoded scRNA-seq libraries using the Chromium Single Cell Library, Gel Bead & Multiplexing Kit (10x Genomics), following the guidelines provided by the manufacturer. In summary, the cells were partitioned into gel beads in an emulsion using the Chromium Controller instrument, followed by cell lysis and reverse transcription of RNA barcodes. The libraries were prepared using the 10x Genomics Chromium Single Cell 3′ Library & Gel Bead Kit v3.1 according to the manufacturer’s prescribed protocol. Sequencing was then performed using an Illumina Nova6000 sequencer to obtain 150 bp paired-end reads.

### scRNA-seq quantification and statistical analysis

The obtained data were imported into R studio and subjected to data normalization, removal of contaminants, and quality control procedures. Cells were filtered based on specific criteria, namely gene counts below 500 and mitochondrial content exceeding 20% using Seurat. Dimensionality reduction and clustering analyses were then carried out using Seurat v3.1.2. Initially, the Normalize Data and Scale Data functions in Seurat were used to standardize and adjust the gene expression data. Subsequently, the Find Variable Features function identified the top 5,000 genes with significant variability for subsequent principal component analysis (PCA). The UMAP algorithm was applied to visualize cells in a two-dimensional space. To annotate cell types within each cluster, we integrated expression profiles of differentially expressed genes with information from relevant literature. The resulting cell type markers were visualized using heatmaps, dot plots, and violin plots generated by the Seurat DoHeatmap/DotPlot/Vlnplot functions, respectively. Doublets expressing markers of different cell types were identified and manually removed.

### Bioluminescence imaging

The transportation of Aβ in brain tissue and optic nerves was quantitatively analyzed using fluorescence and the IVIS Spectrum in vivo imaging system (PerkinElmer) at 30 or 60 min after intracisternal injection.

### MRI

MRI scanning of the mouse head was conducted in the coronal and sagittal planes using a 9.4 T Bruker MR system (BioSpec 94/20 USR; Bruker) following intracisternal administration of Gd-DTPA contrast agent for durations of 10, 20, 30, and 60 min. The MRI system was equipped with a gradient setting of 440 mT/m, an 86-mm volume transfer radio-frequency coil, and a single-channel surface head coil.

### Statistical analysis

Statistical analysis utilized GraphPad Prism 9.5 software (RRID:SCR_002798). Experiments were performed with full blinding and randomization. The normality of data was assessed using the Shapiro–Wilk test, while the homogeneity of variance was evaluated using Brown–Forsythe or F tests. Unpaired/paired two-tailed *t* tests or nonparametric Mann–Whitney U tests or Wilcoxon signed-rank tests (for non-normally distributed data) were employed for comparing two groups. One-way ANOVA or two-way ANOVA with post hoc Tukey tests were conducted for multiple group comparisons. Repeated-measures ANOVA with post hoc Bonferroni tests were applied for analyses involving multiple time points or repeated measurements. *P < 0.05, **P < 0.01, ***P < 0.001; NS indicates no significant difference. Detailed explanations of all statistical methods and corresponding significance levels are available in the figure legends and Table S3.

### Online supplemental material

Fig. S1 shows the multitime point transport of Aβ in the optic nerve. Fig. S2 shows the transport of the contrast agent along the optic nerve. Fig. S3 shows the cellular distribution of AQP4 in the retina and optic nerve. Fig. S4 shows long-range tracing revealed the dissipation pattern of brain-derived Aβ in the retina. Fig. S5 shows no obvious visual impairments in mice after intracerebral injection of exogenous Aβ_1–42_ during 3 days. Table S1 shows studies investigating ocular pathological changes in Alzheimer’s patients. Table S2 shows the antibodies used. Table S3 shows statistical tests for data presented in all figures. Table S4 shows primers used for genotyping of murine strains.

## Supplementary Material

Table S1 shows studies investigating ocular pathological changes in Alzheimer’s patients.

Table S2 shows antibodies used for western blotting, immunofluorescence, and FACS.

Table S3 shows statistical tests for data presented in all figures.

Table S4 shows primers used for genotyping of murine strains.

SourceData F4 is the source file for Fig. 4.

## Data Availability

The raw scRNA-seq datasets of mouse retina (fastq format) have been deposited to NCBI-SRA with the accession number PRJNA1061469.
