# Supplementary material for: Transport of β-amyloid from brain to eye causes retinal degeneration in Alzheimer’s disease
Source: J Exp Med. 2024 Sep 24;221(11):e20240386. doi: 10.1084/jem.20240386 (PMC11448872; doi:10.1084/jem.20240386)
Supplement: Table S1 — shows studies investigating ocular pathological changes in Alzheimer’s patients. [file JEM_20240386_TableS1.docx]

**Table S1. Studies investigating ocular pathological changes in Alzheimer's patients.**

| Tissue | Pathology | Findings | References |
| --- | --- | --- | --- |
| **retina** | Aβ | retinal Aβ plaques in postmortem eyes | (Asanad et al., 2019; Blanks et al., 1989; Koronyo-Hamaoui et al., 2011; Koronyo et al., 2017; Tsai et al., 2014) |
|  |  | amyloid angiopathy | (Blanks et al., 1989) |
|  |  | Aβ accumulation was remarkably evident inside and around mRGCs | (La Morgia et al., 2016) |
|  |  | Aβ associated with blood vessels and occurred in peripheral regions of the superior quadrant and innermost retinal layers | (Koronyo et al., 2017) |
|  |  | not detected | (Ho et al., 2014; Schon et al., 2012) |
|  |  |  |  |
|  | Tau | pTau was increased in the inner and OPL | (den Haan et al., 2018) |
|  |  |  |  |
|  | Structural | RNFL and RGC thickness | (Larrosa et al., 2014; Marziani et al., 2013; Moreno-Ramos et al., 2013) |
|  |  | retinal structural alterations were seen only in the inner layers | (Bayhan et al., 2015) |
|  |  | a significant reduction of RNFL thickness in the nasal superior sector | (Kromer et al., 2014) |
|  |  | a significant thinning for all assessed retinal layers | (Golzan et al., 2017) |
|  |  | a decrease in mRNFL, ONL, and IPL volumes, in preclinical AD | (Santos et al., 2018) |
|  |  | a preferential involvement of the inner retinal layers, especially GCC, in patients with MCI | (Bayhan et al., 2015; Santos et al., 2018) |
|  |  |  |  |
|  | neuronal degeneration | reduction in the number of RGC and in the thickness of the NFL | (Berisha et al., 2007; Hinton et al., 1986; La Morgia et al., 2016; Sadun and Bassi, 1990) |
|  |  | neuronal degeneration in the GCL | (Blanks et al., 1989) |
|  |  |  |  |
|  | vascular abnormalities | narrow veins and decreased retinal blood flow in these veins. | (Berisha et al., 2007; Feke et al., 2015) |
|  |  | altered microvascular network in the retina (narrower retinal venules and a sparser and more tortuous retinal vessels) | (Cheung et al., 2014; Williams et al., 2015) |
|  |  | oxygen saturation in retinal arterioles and venules was statistically increased | (Einarsdottir et al., 2016) |
|  |  | vascular attenuation, increasing standard deviation of vessel widths, reduced complexity of the branching pattern, reduced optimality of the branching geometry and less tortuous venules. | (Frost et al., 2013) |
|  | other | retinal imaging scores are correlated with brain Aβ loads | (Hadoux et al., 2019) |
|  |  | no significant correlation between clinical parameters of AD and parameters of retinal nerve fiber layer thickness. | (Jentsch et al., 2015) |
|  |  |  |  |
| **optic nerve** | Aβ | amyloid angiopathy in the optic nerves | (Blanks et al., 1989) |
|  | Neuronal degeneration | axonal degeneration in the optic nerves | (Hinton et al., 1986) |
|  |  |  |  |
| **lens** | Aβ | Aβ deposits in the lens fiber cells | (Goldstein et al., 2003) |
|  |  | SN region of the human lens | (Kerbage et al., 2013) |
|  |  | not detected | (Ho et al., 2014; Michael et al., 2014; Michael et al., 2013) |
|  |  |  |  |
| **RPE-choroid** | structural | the choroidal thickness was significantly reduced | (Gharbiya et al., 2014; Tsai et al., 2014) |
| Abbreviations: Aβ, β-amyloid mRGCs, melanopsin-expressing retinal ganglion cells; OPL, outer plexiform layers; RNFL, retinal nerve fiber layer; RGC, retinal ganglion cell; mRNFL, macular retinal nerve fiber layer; ONL, outer nuclear layer; IPL, inner plexiform layer; GCC, ganglion cell complex; MCI, mild cognitive impairment; NFL, nerve fiber layer; GCL, ganglion cell layer; AD, alzheimer’s disease; SN, supranucleus; RPE, retinal pigment epithelium. | | | |
