# Supplementary material for: Transport of β-amyloid from brain to eye causes retinal degeneration in Alzheimer’s disease
Source: J Exp Med. 2024 Sep 24;221(11):e20240386. doi: 10.1084/jem.20240386 (PMC11448872; doi:10.1084/jem.20240386)
Supplement: Table S2 — shows antibodies used for western blotting, immunofluorescence, and FACS. [file JEM_20240386_TableS2.docx]

**Table S2. Antibodies used**

Antibodies used for western blotting, immunofluorescence, and FACS

| Antibodies | Source | Catalog number | RRID | Species | Dilution  IF | WB | FACS |
| --- | --- | --- | --- | --- | --- | --- | --- |
| APP | Sigma | sab4300464 | AB_10621836 | rabbit | 1:200 | 1:500 |  |
| AQP4 | Millipore | AB3594 | AB_91530 | rabbit | 1:200 | 1:500 |  |
| Anti-beta Amyloid 1-42 | Abcam | AB201060 | AB_2818928 | rabbit | 1:200 |  |  |
| CD31 | R&D Systems | AF3628 | AB_2161028 | Goat | 1:200 |  |  |
| GAPDH | proteintech | 60004-1-Ig | AB_2107436 | mouse |  | 1:3000 |  |
| GFAP | Millipore | MAB360 | AB_11212597 | mouse |  | 1:1000 |  |
| GFAP | Abcam | ab4674 | AB_304558 | chicken | 1:200 |  |  |
| Iba-1 | Wako | 019-19741 | AB_839504 | rabbit | 1:200 | 1:500 |  |
| Iba-1 | Abcam | Ab5076 | AB_2224402 | goat | 1:200 |  |  |
| Laminin | Sigma | L2020-1MG | N/A | mouse | 1:200 |  |  |
| Laminin | Abcam | AB11575 | AB_298179 | rabbit | 1:200 |  |  |
| Lyve-1 | Abcam | AB33682 | AB_881387 | rabbit | 1:200 |  |  |
| MBP | Abcam | AB7349 | AB_305869 | Rat | 1:200 |  |  |
| NLRP3 | AdipoGen | AG-20B-0014-C100 | AB_2885199 | mouse |  | 1:500 |  |
| Prox1 | Abcam | AB199359 | AB_2868427 | rabbit | 1:200 |  |  |
| PS1 | Sigma | PRS4203 | AB_1855804 | rabbit | 1:200 | 1:500 |  |
| Rhodopsin | Abcam | AB190307 | AB_2747769 | mouse | 1:200 |  |  |
| RPE65 | Abcam | AB231782 | AB_2922412 | rabbit | 1:200 |  |  |
| TNFα | Abcam | AB6671 | AB_305641 | rabbit |  | 1:500 |  |
| ZO-1 | Invitrogen | 33-9100 | AB_2533147 | mouse | 1:200 |  |  |
| β-III tubulin | Abcam | AB52623 | AB_869991 | rabbit | 1:200 |  |  |
| 6E10 | Biolegend | 803001 | AB_2564653 | mouse | 1:200 | 1:500 |  |
| 4G8 | Biolegend | 800712 | AB_2734548 | mouse | 1:200 |  |  |
| Peanut Agglutinin (PNA) | Vector Laboratories | FL-1071 | AB_2315097 |  | 1:200 |  |  |
| Griffonia Simplicifolia Lectin 1(GSL I) lsolectin B4 | Vector Laboratories | DL-1207 | AB_2336415 |  | 1:100 |  |  |
| Griffonia Simplicifolia Lectin 1(GSL I) lsolectin B4 | Vector Laboratories | FL-1201 | AB_2314663 |  | 1:100 |  |  |
| Mouse (G3A1) mAb IgG1 Isotype Control | Cell Signaling Technology | 5415S | AB_10829607 | mouse | 1:200 |  |  |
| Rabbit (DA1E) mAb IgG XP® Isotype Control | Cell Signaling Technology | 3900S | AB_1550038 | rabbit | 1:200 |  |  |
| [Horseradish enzyme labeled goat anti-mouse IgG (H+L)](https://scicrunch.org/resources/data/record/nif-0000-07730-1/AB_2747415/resolver?q=*&l=*&filter%5b%5d=Catalog%20Number:ZB-2305&i=2828892) | ZSGB-BIO | ZB-2305 | AB_2747415 | goat |  | 1:2000 |  |
| [Horseradish enzyme labeled goat anti-rabbit IgG (H+L)](https://scicrunch.org/resources/data/record/nif-0000-07730-1/AB_2747412/resolver?q=*&l=*&filter%5b%5d=Catalog%20Number:ZB-2301&i=2828893) | ZSGB-BIO | ZB-2301 | AB_2747412 | goat |  | 1:2000 |  |
| AF488-conjugated anti-mouse IgG | ThermoFisher | A21202 | AB_141607 | donkey | 1:1000 |  |  |
| AF488-conjugated anti-rabbit IgG | ThermoFisher | A21206 | AB_2535792 | donkey | 1:1000 |  |  |
| AF488-conjugated anti-chicken IgG | Abcam | AB150169 | AB_141607 | goat | 1:1000 |  |  |
| AF488-conjugated anti-rat IgG | Abcam | AB150153 | AB_2737355 | donkey | 1:1000 |  |  |
| AF555-conjugated anti-rabbit IgG | ThermoFisher | A31572 | AB_162543 | donkey | 1:1000 |  |  |
| AF555-conjugated anti-mouse IgG | ThermoFisher | A31570 | AB_2536180 | donkey | 1:1000 |  |  |
| AF555-conjugated anti-goat IgG | ThermoFisher | A21432 | AB_2535853 | donkey | 1:1000 |  |  |
| AF647-conjugated anti-rabbit IgG | ThermoFisher | A32795TR | AB_2866496 | donkey | 1:1000 |  |  |
| AF647-conjugated anti-rat IgG | Abcam | AB150155 | AB_2813835 | donkey | 1:1000 |  |  |
| CD31 | Biolegend | 102416 | AB_493410 | rat |  |  | 1:200 |
| CD45 | ThermoFisher | 11-0451-82 | AB_465050 | rat |  |  | 1:100 |
| CD73 | ThermoFisher | 12-0731-82 | AB_763513 | rat |  |  | 1:150 |
