# Supplementary material for: Transport of β-amyloid from brain to eye causes retinal degeneration in Alzheimer’s disease
Source: J Exp Med. 2024 Sep 24;221(11):e20240386. doi: 10.1084/jem.20240386 (PMC11448872; doi:10.1084/jem.20240386)
Supplement: Table S3 — shows statistical tests for data presented in all figures. [file JEM_20240386_TableS3.docx]

**Table S3. Statistical tests for data presented in all figures**

| Figures | | Sample group | Statistic method |
| --- | --- | --- | --- |
| Fig. 2 | Fig. 2 B | 6E10 expression in retina: WT vs 5×FAD | Unpaired two-tailed T test |
|  | Fig. 2 E | 6E10 expression in optic nerve: WT vs 5×FAD | Unpaired two-tailed T test |
|  | Fig. 2 G | Aβ_1-40_ concentration: WT vs 5×FAD | Unpaired two-tailed T test |
|  | Fig. 2 H | Aβ_1-42_ concentration: WT vs 5×FAD | Unpaired two-tailed T test |
| Fig. 3 | Fig. 3 B | Number of highly reflective spots: WT vs 5×FAD | Mann-Whitney test |
|  | Fig. 3 C | Retinal thickness: WT vs 5×FAD | Repeated measures ANOVA with Bonferroni post hoc tests |
|  | Fig. 3 E | Time spent in the dark box: WT vs 5×FAD | Mann-Whitney test |
|  | Fig. 3 F | Number of entries into the dark box: WT vs 5×FAD | Mann-Whitney test |
|  | Fig. 3 H | Bouts of nutation movement: WT vs 5×FAD | Repeated measures ANOVA with Bonferroni post hoc tests |
|  | Fig. 3 I | Time of nutation movement: WT vs 5×FAD | Repeated measures ANOVA with Bonferroni post hoc tests |
|  | Fig. 3 K | RPE65 expression: WT vs 5×FAD | Unpaired two-tailed T test |
|  | Fig. 3 M | PNA and Rhodopsin expression: WT vs 5×FAD | Unpaired two-tailed T test |
|  | Fig. 3 O | Retinal thickness: WT vs 5×FAD | Unpaired two-tailed T test |
| Fig. 4 | Fig. 4 B | APP and PS1 expression in brain: healthy controls vs AD donors | Unpaired two-tailed T test |
|  | Fig. 4 D | PS1 expression in brain: WT vs 5×FAD | One-way ANOVA with post hoc Tukey tests |
|  | Fig. 4 E | PS1 expression in brain: WT vs 5×FAD | One-way ANOVA with post hoc Tukey tests |
|  | Fig. 4 H | APP expression in retina: WT vs 5×FAD, healthy controls vs AD donors | Unpaired two-tailed T test |
|  | Fig. 4 I | PS1 expression in retina: WT vs 5×FAD, healthy controls vs AD donors | Unpaired two-tailed T test |
|  | Fig. 4 K | APP and PS1 expression in brain and retina: WT vs 5×FAD | One-way ANOVA with post hoc Tukey tests |
|  | Fig. 4 N | APP expression in retinal endothelial cells and pericytes: WT vs 5×FAD | Unpaired two-tailed T test |
|  | Fig. 4 P | PS1 expression in retinal ganglion cells and macrophages/microglia: WT vs 5×FAD | Unpaired two-tailed T test |
| Fig. 7 | Fig. 7 B | Time of nutation movement: Vehicle-Day 7 vs Aβ-Day 7, Vehicle -Day 15, Aβ-Day 15 | Repeated measures ANOVA with Bonferroni post hoc tests |
|  | Fig. 7 C | Bouts of nutation movement: Vehicle-Day 7 vs Aβ-Day 7, Vehicle -Day 15, Aβ-Day 15 | Repeated measures ANOVA with Bonferroni post hoc tests |
|  | Fig. 7 E | Retinal thickness: Vehicle-Day 7 vs Aβ-Day 7, Vehicle -Day 15, Aβ-Day 15 | One-way ANOVA with post hoc Tukey tests |
|  | Fig. 7 F | Retinal thickness: Vehicle-Day 7 vs Aβ-Day 7, Vehicle -Day 15, Aβ-Day 15 | Repeated measures ANOVA with Bonferroni post hoc tests |
|  | Fig. 7 J | RPE65 expression: Day 3 vs Day 7, Day 15 | One-way ANOVA with post hoc Tukey tests |
|  | Fig. 7 K | TUJ1 expression: Day 3 vs Day 7, Day 15 | One-way ANOVA with post hoc Tukey tests |
|  | Fig. 7 L | Rhodopsin expression: Day 3 vs Day 7, Day 15 | One-way ANOVA with post hoc Tukey tests |
| Fig. 8 | Fig. 8 B | Fluorescence intensity in long-term tracing experiments: WT vs KO | Unpaired two-tailed T test |
|  | Fig. 8 F | Bouts of nutation movement: WT DMSO group vs WT Aβ group, KO DMSO group, KO Aβ group | Repeated measures ANOVA with Bonferroni post hoc tests |
|  | Fig. 8 G | Time of nutation movement: WT DMSO group vs WT Aβ group, KO DMSO group, KO Aβ group | Repeated measures ANOVA with Bonferroni post hoc tests |
|  | Fig. 8 I | Retinal thickness: WT DMSO group vs WT Aβ group, KO DMSO group, KO Aβ group | Repeated measures ANOVA with Bonferroni post hoc tests |
|  | Fig. 8 J | Retinal thickness: WT DMSO group vs WT Aβ group, KO DMSO group, KO Aβ group | Two-way ANOVA with post hoc Tukey tests |
|  | Fig. 8 L | RPE65 expression: WT DMSO group vs WT Aβ group, KO DMSO group, KO Aβ group | Two-way ANOVA with post hoc Tukey tests |
|  | Fig. 8 M | TUJ1 expression: WT DMSO group vs WT Aβ group, KO DMSO group, KO Aβ group | Two-way ANOVA with post hoc Tukey tests |
|  | Fig. 8 N | Rhodopsin expression: WT DMSO group vs WT Aβ group, KO DMSO group, KO Aβ group | Two-way ANOVA with post hoc Tukey tests |
| Fig. 9 | Fig. 9 E | AQP4 polarization index of retinal capillaries: WT vs 5×FAD, Aged | One-way ANOVA with post hoc Tukey tests |
| Fig. S1 | Fig. S1 D | Aβ transportation distance: 30 minutes vs 60 minutes after injection | Unpaired two-tailed T test |
|  | Fig. S1 G | Area covered in short-term tracing experiments: 30 min vs 60 min | Unpaired two-tailed T test |
|  | Fig. S1 J | Aβ fluorescence intensity: Aβ injection vs NS injection | Two-way ANOVA with post hoc Tukey tests |
|  | Fig. S1 K | Aβ fluorescence intensity: optic nerve ligation vs control | Unpaired two-tailed T test |
| Fig. S3 | Fig. S3 J | Aβ fluorescence intensity: WT vs KO | Two-way ANOVA with post hoc Tukey tests |
|  | Fig. S3 L | Aβ transportation area: WT vs KO | Unpaired two-tailed T test |
|  | Fig. S3 N | Aβ transportation distance: WT vs KO | Unpaired two-tailed T test |
| Fig. S5 | Fig. S5 B | Time of nutation movement: Vehicle-Day 3 vs Aβ-Day 3 | Repeated measures ANOVA with Bonferroni post hoc tests |
|  | Fig. S5 C | Bouts of nutation movement: Vehicle-Day 3 vs Aβ-Day 3 | Repeated measures ANOVA with Bonferroni post hoc tests |
|  | Fig. S5 E | Retinal thickness: Vehicle-Day 3 vs Aβ-Day 3 | Unpaired two-tailed T test |
|  | Fig. S5 F | Retinal thickness: Vehicle-Day 3 vs Aβ-Day 3 | Repeated measures ANOVA with Bonferroni post hoc tests |
