# Supplementary material for: Transport of β-amyloid from brain to eye causes retinal degeneration in Alzheimer’s disease
Source: J Exp Med. 2024 Sep 24;221(11):e20240386. doi: 10.1084/jem.20240386 (PMC11448872; doi:10.1084/jem.20240386)
Supplement: Table S4 — shows primers used for genotyping of murine strains. [file JEM_20240386_TableS4.docx]

**Table S4. Primers used for genotyping of murine strains**

| **Primers** | **Sequence (5’-3’)** | **Product size** |
| --- | --- | --- |
| APP | AGG ACT GAC CAC TCG ACC AG | T: 377 bp  W: none |
|  | CGG GGG TCT AGT TCT GCA T |  |
| PSEN1 | AAT AGA GAA CGG CAG GAG CA | T: 680 bp  W: none |
|  | GCC ATG AGG GCA CTA ATC AT |  |
| AQP4 | ACC ATA AAC TGG GGT GGC TCA G | T: 320 bp  W: 240 bp |
|  | TAG AGG ATG CCG GCT CCA ATG A |  |
|  | CAC CGC TGA ATA TGC ATA AGG CA |  |
