# Supplementary figures and images for: Transport of β-amyloid from brain to eye causes retinal degeneration in Alzheimer’s disease
Source: J Exp Med. 2024 Sep 24;221(11):e20240386. doi: 10.1084/jem.20240386 (PMC11448872; doi:10.1084/jem.20240386)

Figure 4J

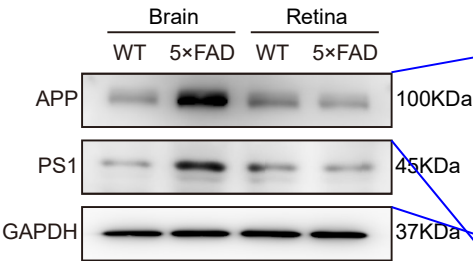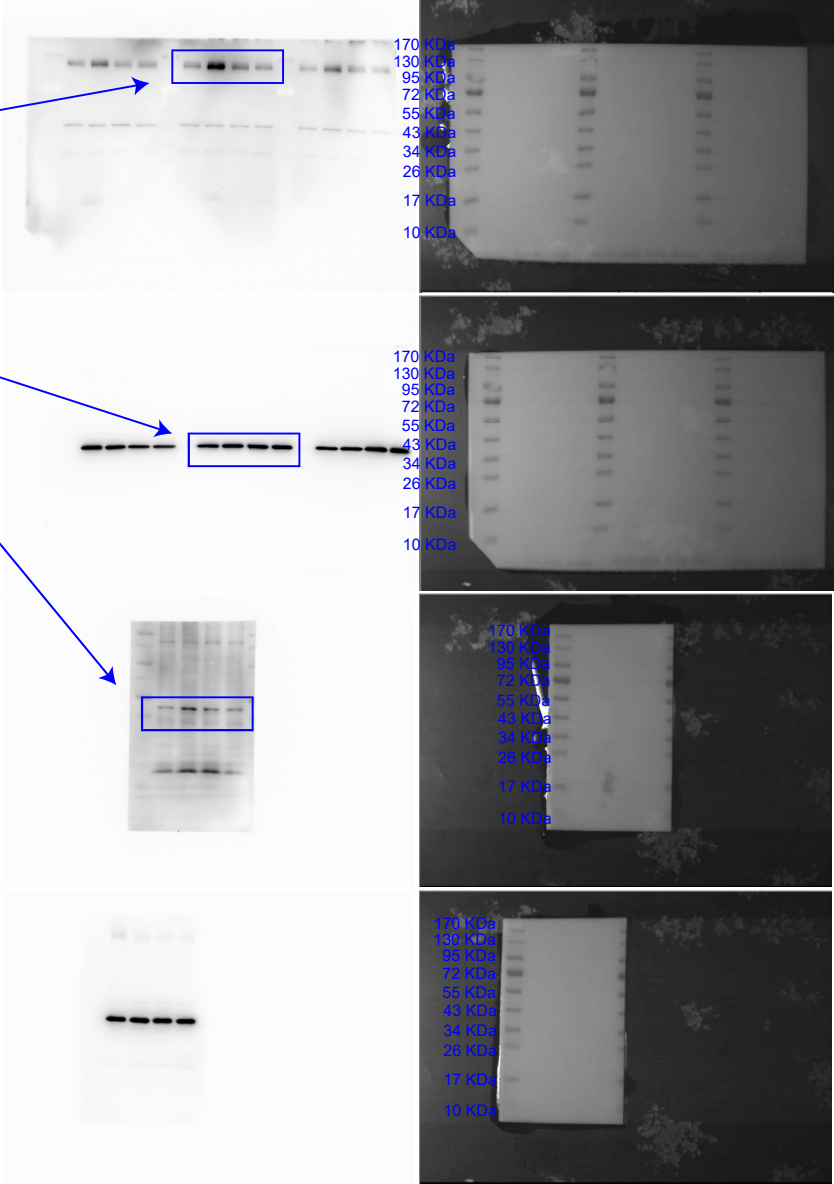

Supplement: SourceData F4 — is the source file for Fig. 4. [file JEM_20240386_SourceDataF4.pdf]
